# Clay and Microsilica Additives’ Effect on the Properties and Structure of Injectable Cement–Clay Mortars for Soil Consolidation

**DOI:** 10.3390/ma19173611

**Published:** 2026-08-25

**Authors:** Evgenii M. Shcherban’, Sergey A. Stel’makh, Alexey N. Beskopylny, Diana M. Shakhalieva, Andrei Chernil’nik, Natalya Shcherban’, Valery Varavka, Yasin Onuralp Özkılıç

**Affiliations:** 1 Department of Engineering Geometry and Computer Graphics, Don State Technical University, 344003 Rostov-on-Don, Russia; etsherban@donstu.ru; 2 Department of Unique Buildings and Constructions Engineering, Don State Technical University, 344003 Rostov-on-Don, Russia; sstelmah@donstu.ru (S.A.S.); achernilnik@donstu.ru (A.C.); ndocenko@donstu.ru (N.S.); 3 Department of Transport Systems, Faculty of Roads and Transport Systems, Don State Technical University, 344003 Rostov-on-Don, Russia; 4 Department of Design, Don State Technical University, 344003 Rostov-on-Don, Russia; delshaeva@donstu.ru; 5 Research and Education Center “Materials”, Don State Technical University, Gagarin Sq. 1, 344003 Rostov-on-Don, Russia; vvaravka@donstu.ru; 6 Department of Civil Engineering, Faculty of Engineering, Necmettin Erbakan University, Konya 42000, Turkey; 7 Department of Technical Sciences, Western Caspian University, Baku 1001, Azerbaijan

**Keywords:** concrete, injection cement–clay mortars, compressive strength, flexural strength, clay, microsilica

## Abstract

The potential of clay as a replacement for Portland cement in the manufacture of injection cement–clay mortars (ICCMs) for soil stabilization is examined in this investigation. The objective of this study is to produce environmentally friendly injection-molded mortars for soil stabilization based on Portland cement (PC) and clay (C). Experimental ICCMs with C contents ranging from 0% to 50% without the addition of microsilica (MS) and ICCMs with C contents ranging from 0% to 50% and 2% MS were produced. The evaluation included the density, water segregation, and cone spread diameter of fresh ICCMs, alongside the density, flexural strength, and compressive strength of hardened ICCMs. The findings indicated that as C content rose from 0% to 50%, fresh mortars experienced a decrease in density, flowability, and water segregation. Hardened mortars exhibited reduced density, compressive strength, and flexural strength as C content increased. Modifying mortars with MS has a positive effect on their strength properties. The reduction in flexural and compressive strength for mortars with 50% C was 54.2% and 60.1%, respectively, while for similar mortars with 2% MS, the reduction in strength was 47.9% and 51.8%, respectively. ICCM soil stabilization compositions modified with MS are the most effective in comparison with similar compositions without MS and have a homogeneous structure with pores, microcracks, and hydration reaction product zones. The optimal ratios of raw components for the production of ICCMs for soil stabilization were determined: a water–solid ratio of 0.6, PC content from 90% to 50%, C content from 10% to 50%, and an MS content of 2% of the dry component weight. This research contributes to sustainable development by reducing CO_2_ emissions per 1 m^3^ of mixture production by up to 47.8% and by using raw materials rationally.

## 1. Introduction

Soft and unstable soils pose a serious challenge to civil, industrial, and road construction, as they have poor bearing capacity and are easily eroded, which leads to structural instability and premature failure [1,2]. To improve soil stability and increase its bearing capacity, various modern soil stabilization methods are used, which mainly involve the introduction of cement and geopolymer mortars with various modifying additives into the soil [3,4]. The stabilization of alluvial pavement with ground granulated blast furnace slag (GGBS) and fly ash (FA) increases the bearing capacity and dynamic modulus of the soil [5]. The inclusion of silicon oxide (SiO_2_) and calcium carbonate (CaCO_3_) nanoparticles in a cement-based soil stabilizer improves its strength properties [6]. Combining cement with coal enrichment waste enhances soil’s mechanical properties and decreases its porosity [7]. Plasticity and swelling potential in heaving soils are decreased by using sodium chloride (NaCl) and lime slurry [8]. The properties of sulfate-saline soils were improved by consolidation using a composite system based on guar gum (GA) and Portland cement [9]. To increase the compressive strength from 0.71 kg/cm^2^ to 3.5–4.3 kg/cm^2^ after 28 days of curing, collapsible soils are reinforced with environmentally friendly supplements (lime, rice husk ash, polypropylene fibers, eggshell powder, nanosilica) [10]. A mixture of silty soils with GGBS and FA and an alkaline activator solution makes it possible to obtain a composite with a compressive strength of 12.3 MPa [11]. The introduction of cement and 15% of boron waste increases the soil density and its bearing capacity [12]. Geopolymer soil stabilization increases soil stiffness by up to 270% [13]. The introduction of 5% iron ore waste improves the mechanical properties of dispersed soils [14]. The stabilization of heaving foundation soils with biochar from rice husks significantly improves their mechanical properties and reduces shrinkage [15]. Sandy–clayey soils stabilized with a biopolymer based on corn starch and nanosilica particles demonstrated a significant increase in compressive strength of up to 465% [16]. A mixture of high-carbon ferrochrome slag and GGBS increases the frost resistance of saline soils [17]. Effective geopolymer materials from a mixture of various wastes and by-products, designed to improve the properties and stabilize soft soils, are being developed by many researchers [18,19,20].

For stabilizing soft soils in difficult geological settings, the jet grouting method is highly effective. This method involves introducing specialized cement-based solutions into the soil to enhance its mechanical characteristics. In construction, this technique is most often used to strengthen slopes and building foundations [21]. Cement, cement–clay, and geopolymer mortars with various additives are used for this method. Developments have been made in multifunctional, environmentally friendly cement mortars with the addition of red mud, phosphogypsum, and metakaolin for the jet grouting of water-saturated sandy soils [22]. The optimal combination of cement, fly ash, bentonite, and a superplasticizing additive allows for the production of effective mortars for geotechnical and underground construction [23]. Substituting part of the cement with 50% GGBFS in the formulation of jet grouting mortars improves their flowability and strength properties [24]. Laboratory tests of geopolymer systems designed for the jet grouting of soils confirm the effectiveness of the proposed solutions, which increase the bearing capability of soil by 5.5 times [25]. Stabilizing solutions based on cement, clay, and the addition of calcium nano-carbonate demonstrate good efficiency in the injection of aquiferous soils [26]. Studies on the jet grouting process’s impact on soil, alongside the influence of pressure and mortar consumption on soil concrete strength, showed substantial improvements in soil properties, including increased uniaxial compressive strength and enhanced adhesion and friction angle [27]. Columns manufactured by jet grouting in less water-saturated sandy soils had up to 50% higher strength properties than columns in fully saturated soils [28]. Jet grouting significantly increases the shear strength of sliding soil and strengthens landslide soils [29]. In general, the jet cementation method is quite often used for the production of columns and demonstrates good efficiency [30,31,32].

Currently, the production of highly penetrating injection mortars for soil stabilization using jet grouting is a pressing issue and combines an effective engineering solution with sustainable development principles [33,34]. The design of injection cement–clay mortars (ICCMs) for soil stabilization is accompanied by technological complexities associated with the compatibility of components such as cement and clay [35,36]. A crucial point is that mortars for jet grouting must satisfy regulatory standards for mobility and strength [37]. Including clay as a raw material by replacing part of Portland cement will allow for savings of a significant portion of the most expensive component [38]. Using alternative materials instead of Portland cement supports sustainable development goals through reduced carbon emissions and wise raw material management [39,40,41,42]. The scientific novelty of this study lies in the development of new cement- and clay-based mortars with the addition of microsilica for soil stabilization using jet grouting and the discovery of new relationships between the mortar properties and structure and formulation parameters. For soil stabilization, this study aims to develop environmentally friendly injection cement–clay mortar formulations. The main objectives of the study are:

– manufacture experimental ICCM compositions for stabilizing soils with varying clay contents and similar formulations additionally modified with microsilica;
– Determine the properties of fresh (density, water segregation, and cone diameter) and hardened ICCMs (density, compressive strength, and flexural strength);
– Investigate the microstructural features of hardened ICCMs using scanning electron microscopy (SEM) and energy diffraction X-ray spectroscopy (EDS);
– Conduct analysis of variance (ANOVA) on the experimental data to determine the characteristics of the ICCMs, evaluate the optimal composition selection, and assess the compliance of their actual values with regulatory requirements.

## 2. Materials and Methods

### 2.1. Materials

To create ICCMs for soil stabilization, the following raw materials were used: Portland cement CEM I 42.5 H (PC) (CEMROS, Stary Oskol, Russia); microsilica (MS) (NLMK, Lipetsk, Russia); and clay (C) (Tarasovskoye mineral deposit, Tarasovsky, Russia).

The chemical composition and main properties of the ICCMs, as provided by the manufacturer, are presented in Table 1.

The chemical composition and main properties of MS, as specified in the manufacturer’s documentation, are displayed in Table 2.

**Figure 1:**
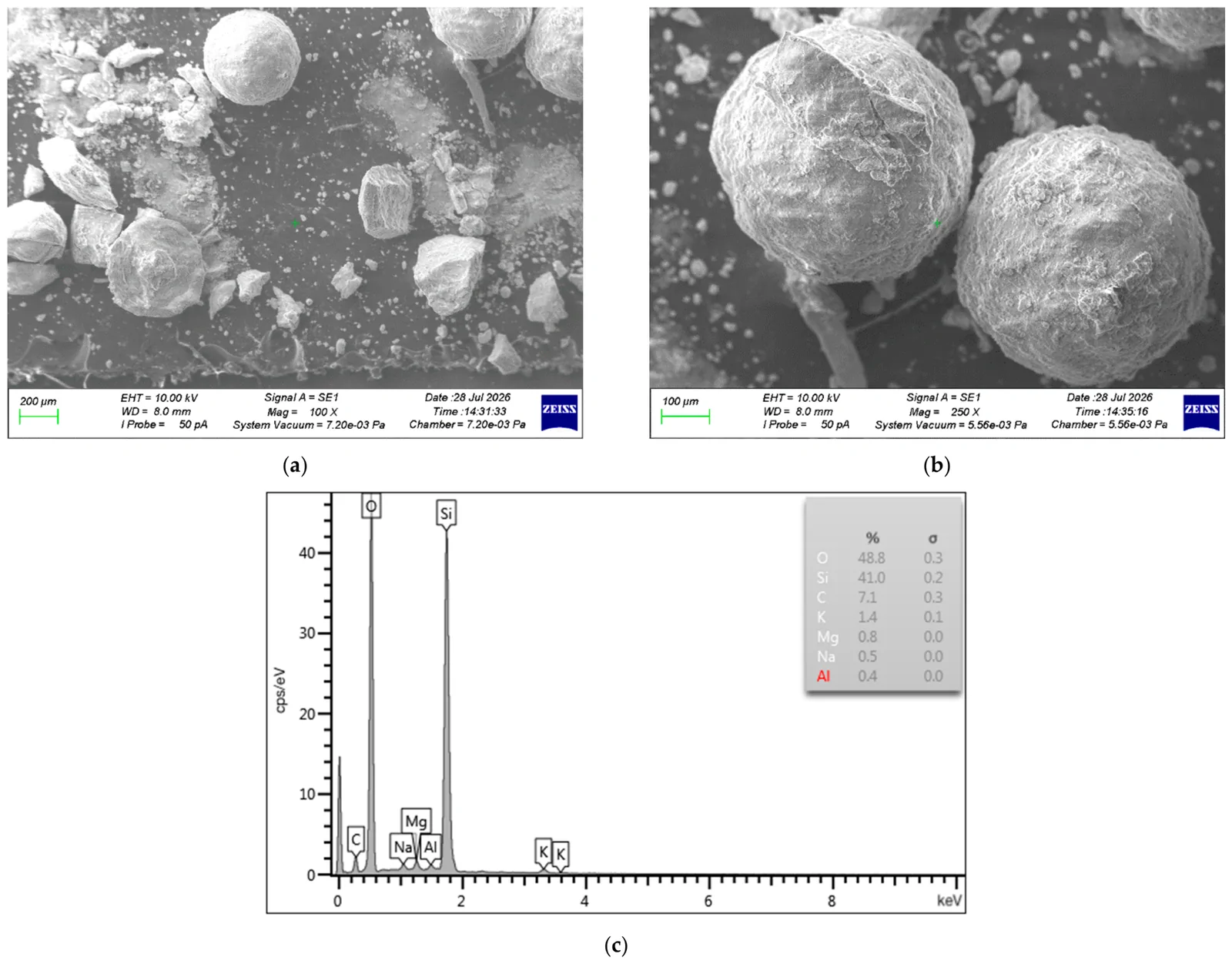
MS: (**a**) at 100× magnification; (**b**) with a magnification of 250×; (**c**) Spectrum 1.

Figure 1 shows the results of SEM and EDS MS analyses. SEM and EDS MS analysis was performed on a Zeiss EVO MA 18 scanning electron microscope (Carl Zeiss Microscopy Ltd., Cambourne, UK) equipped with an X-Max 50N (Oxford Instruments, High Wycombe, UK) energy dispersive spectrometer (EDS) integrated into the electron microscope.

MS particles are characterized by a spherical shape, and irregularly shaped particles are also found. Spectrum 1 shows the following chemical elements: O, Si, C, K, Mg, Na, and Al in amounts of 48.8%, 41.0%, 7.1%, 1.4%, 0.8%, 0.5%, and 0.4%, respectively.

The supplier’s documents provide the chemical composition and basic properties of C, which are displayed in Table 3.

**Figure 2:**
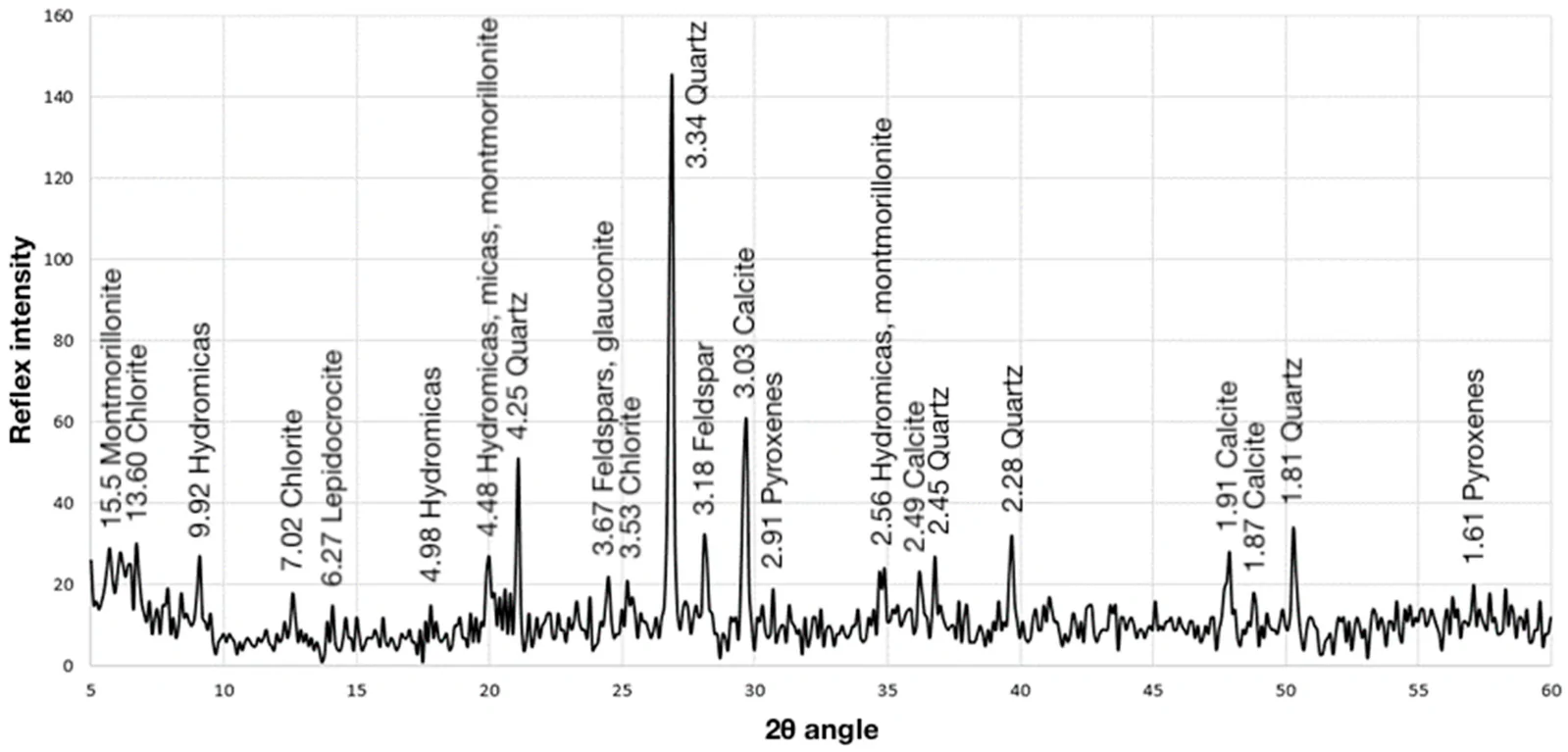
X-ray diffraction pattern of clay.

Radiograph C is shown in Figure 2.

The clay has the following mineral composition: kaolinite—3.0%; hydromicas and micas—21.80%; montmorillonite—24.45%; feldspars—1.0%; carbonates—17.23%; and accessory minerals—1.36%.

**Figure 3:**
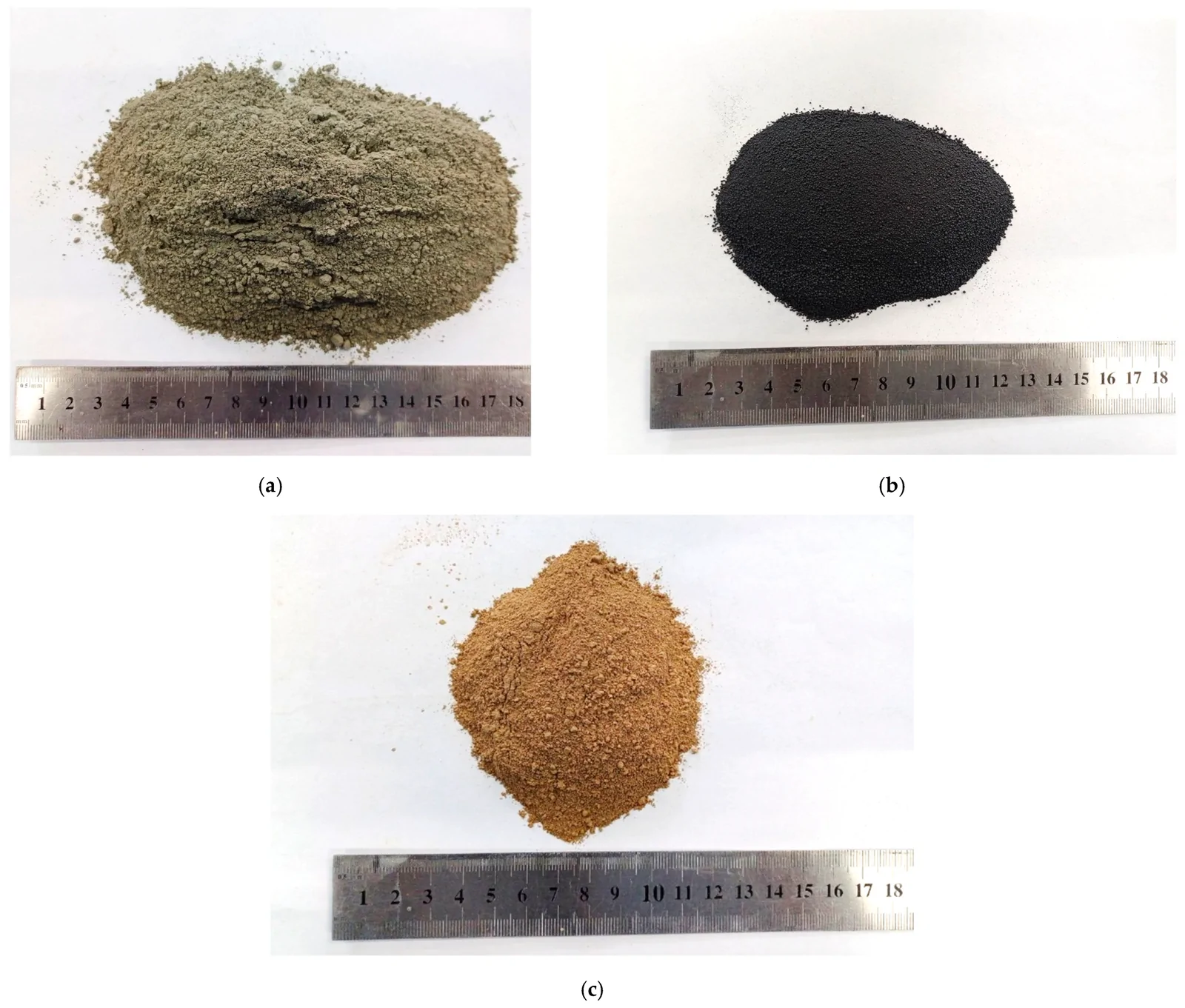
Raw materials: (**a**) PC; (**b**) MS; (**c**) C.

Figure 3 displays the visual features of the original materials.

**Figure 4:**
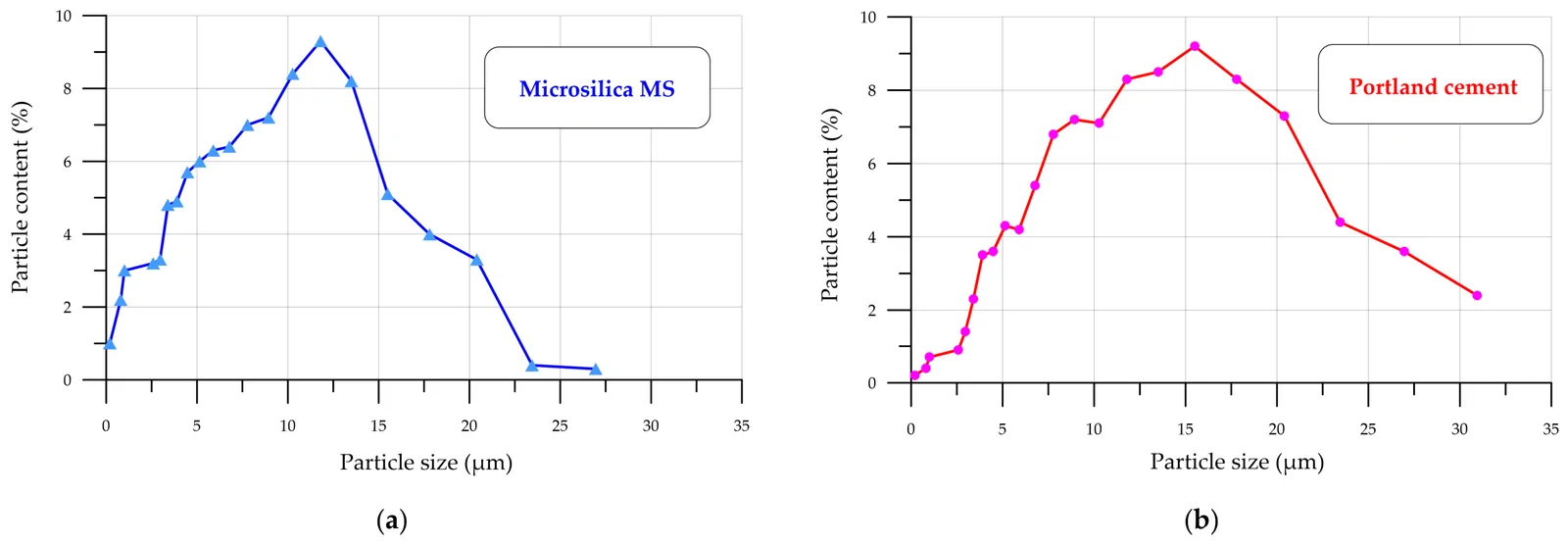
Particle size distribution curves: (**a**) MS; (**b**) PC.

Figure 4 displays the size distribution curves for PC and MS particles.

The proportion of MS particles up to 5 µm is 34.1%, and that from 5 µm to 20 µm is 65.2%. The majority of PC particles (89.6%) are in the size range from 0.2 µm to 20.4 µm.

See Table 4 for the clay particle size distribution.

The majority of clay particles (60.45%) are smaller than 1 μm. Therefore, this clay is classified as a highly dispersed raw material.

### 2.2. Methods

ICCM compositions with varying clay and MS additive contents are presented in Table 5. Clay replaced a portion of the PC, with percentages from 0% to 50% in 10% steps. ICCMs for soil stabilization were prepared without the use of a plasticizing additive in order to evaluate the actual effect of additives C and MS on the mobility parameters of fresh solutions. MS was added as a modifying additive at a rate of 2% of the Portland cement and clay mixture weight. Preliminary experimental studies were conducted to determine the optimal MS additive amount, and the most appropriate MS content was determined. Higher MS dosages resulted in increased water demand and decreased workability of cement–clay mortars. The water-to-binder ratio for all experimental ICCM compositions was maintained at 0.6.

The preparation of the experimental ICCM compositions included the following main steps.

Stage 1. Before use, the clay was sieved through a #2 sieve to remove impurities. The material was then ground by hand using a mortar and pestle and subsequently dried to a consistent weight in a ShS-80-01 MK SPU laboratory drying oven (manufactured by Smolensk SKTB SPU in Smolensk, Russia) at a temperature between 40 and 50 °C. The clay was then soaked in half the mixing water for 24 h. After soaking, the clay was mixed with water for 10 min at a speed of 1000–1200 rpm to obtain a homogeneous solution.

Stage 2. Following the recipe, the raw materials were portioned and blended. PC and MS, along with the remaining water, were combined with the clay sequentially. The entire solution was mixed to a homogeneous consistency and poured into metal molds.

Stage 3. ICCM samples were cured in molds for 2 days. The specimens were then extracted from their molds and stored for the next 26 days in a laboratory environment with a temperature of 20 ± 2 °C and a relative humidity of 60 ± 5%.

**Figure 5:**
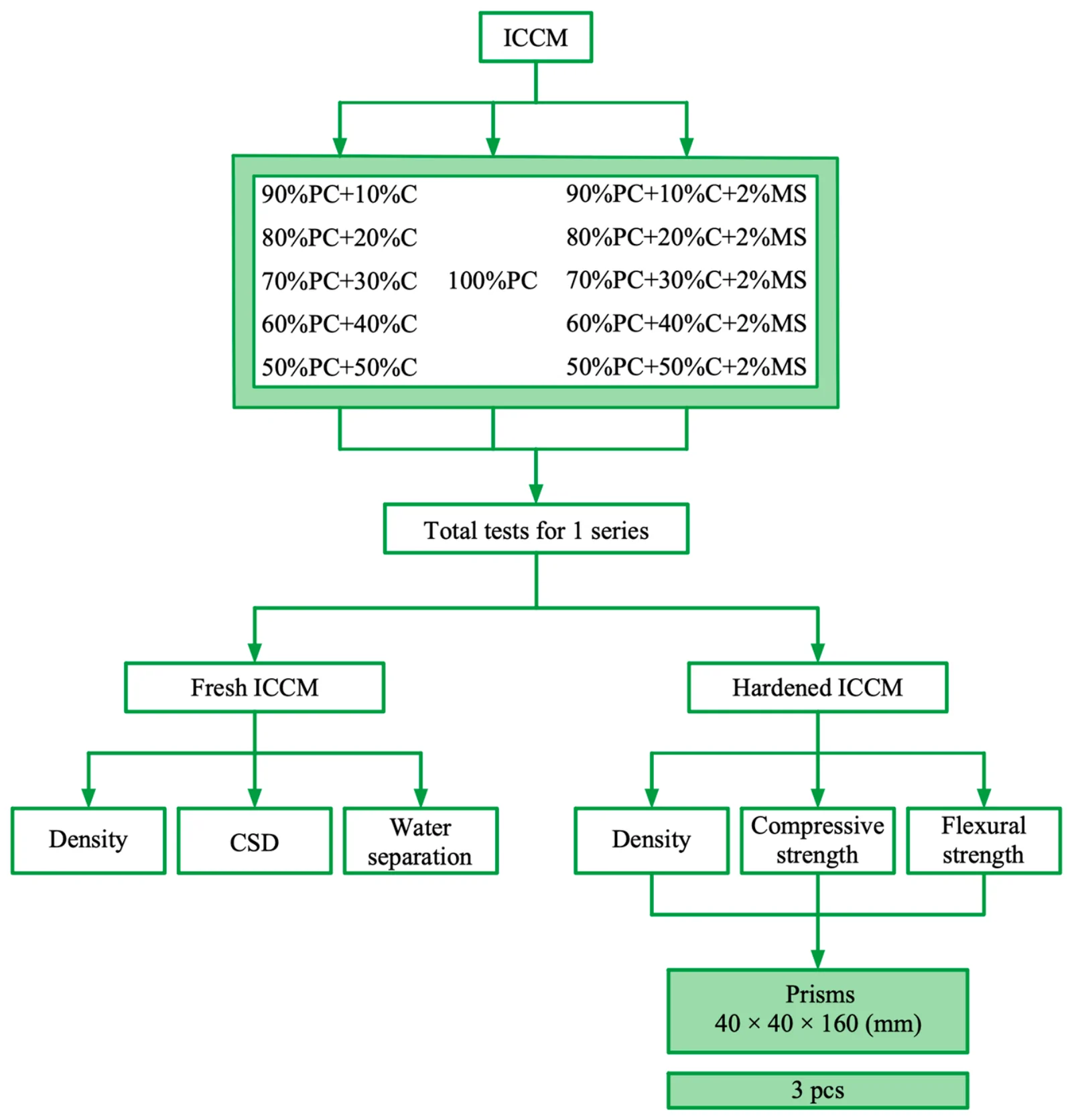
Research program (CSD—cone spread diameter).

Figure 5 illustrates the experimental program for ICCMs.

A total of 11 ICCM compositions were prepared, with three prism-shaped specimens for each of them being fabricated for density and bending tests, which were then used to produce six semi-prism specimens for compression testing. A total of 33 prism-shaped specimens were fabricated, yielding 66 semi-prism-shaped specimens. One series is 3 prism samples and 6 half-prism samples obtained from these 3 prisms. The 100PC compound serves as the control and reference point. Each compound was subjected to six laboratory tests: fresh ICCMs (Test 1: density; Test 2: cone flow diameter; Test 3: water bleeding) and hardened ICCMs (Test 4: density; Test 5: flexural strength; Test 6: compressive strength).

The density, cone flow diameter, and water segregation properties of the ICCMs were determined using the methods in Refs. [43,44]. To determine the density, fresh ICCM was poured into a pre-prepared 100 cm^3^ metal pycnometer, which was then closed with a lid. Excess ICCM was removed with a damp cloth, and the pycnometer containing the mixture was weighed. The ICCM density was calculated using Formula (1) (Table 6). The result of the calculation was rounded to 0.01 g/cm^3^. The determination of the ICCM’s cone flow diameter was conducted in the following manner. Initially, the cone mold was set on a flat surface and then filled to the brim with new mortar. After the excess mortar was removed, the cone was given a sharp upward pull. Measurements of the cone’s spread diameter were taken with a ruler along two perpendicular lines. The CSD was calculated by averaging the two measurements. Fresh ICCMs’ water separation was assessed using the criteria from the method in Ref. [44]. A 500 cm^3^ glass cylinder was filled with fresh mortar, and its actual volume was recorded. Then, the cylinder with the mortar was kept motionless for 2 h. After this period, the volume of the settled mortar was recorded. Water separation was calculated using Formula (2) (Table 6). The results of two parallel tests, run at the same time, were averaged to determine water separation, then rounded to one decimal place.

**Figure 6:**
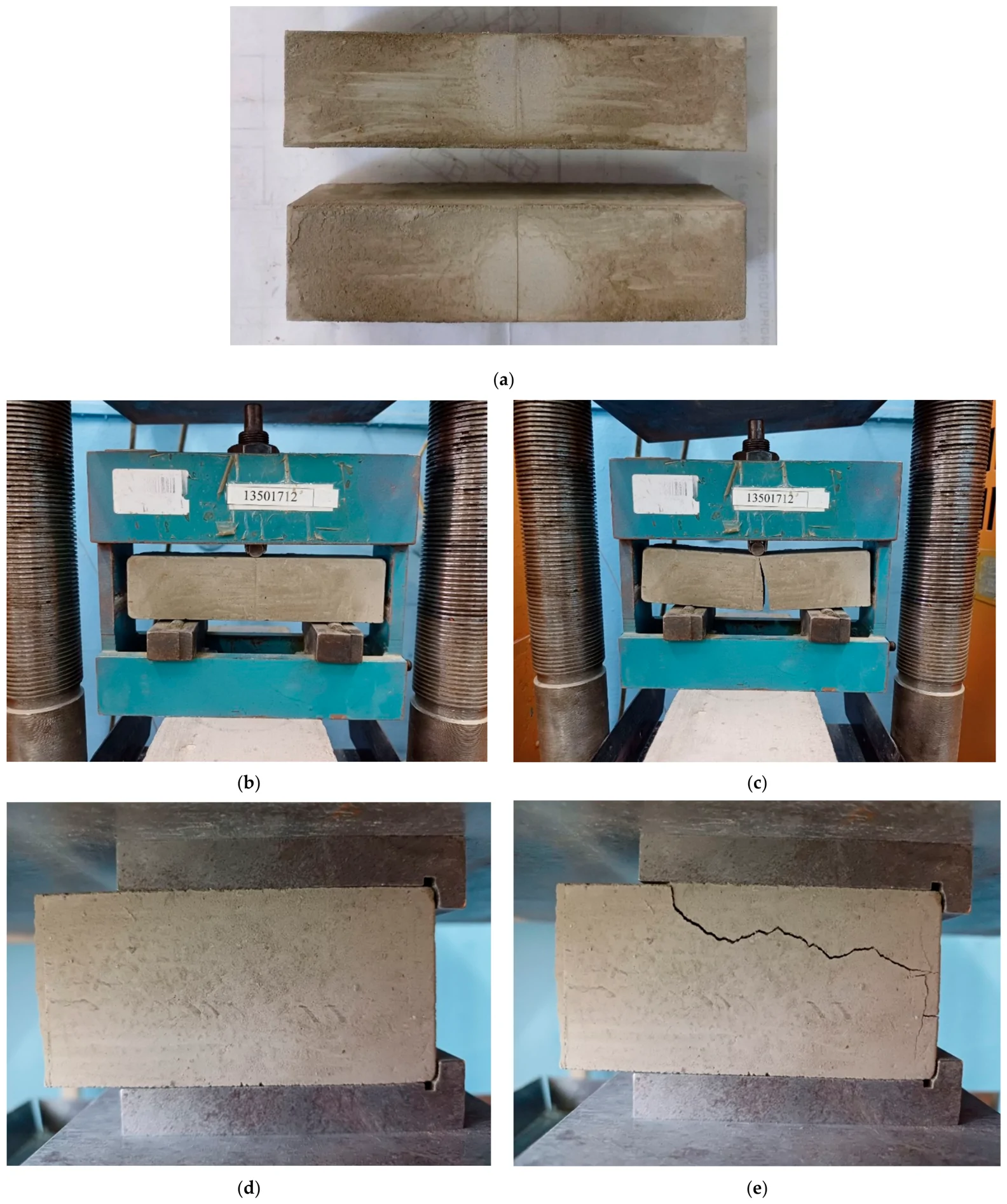
ICCM soil stabilization tests: (**a**) ICCM samples; (**b**) prism specimen before failure; (**c**) prism specimen after failure; (**d**) semi-prism specimen before failure under compression; (**e**) semi-prism specimen after failure.

The approach detailed in Ref. [45] was employed to obtain the density of hardened ICCMs. The geometric parameters of the experimental solution samples were measured and then weighed. Formula (3) from Table 6 was used to determine the density with 1 kg/m^3^ accuracy. The density for each set of samples was determined by averaging the test outcomes of all three samples within that set. According to the requirements of Ref. [43], the compressive and flexural strengths of hardened ICCMs were measured. Initially, 40 × 40 × 160 mm beam samples were installed in a bending test apparatus, and force was applied at a rate of 50 ± 10 N/s. Then, the resulting semi-prism samples were installed in pressure plates to transfer the load. The semi-prism samples with plates were installed in a press, and the load was applied. Formula (4) from Table 6 was used to determine the flexural strength, with a precision of 0.1 MPa. The measurement result was determined by calculating the arithmetic mean of the test results for three prism specimens. Formula (5) from Table 6 was employed to calculate compressive strength, achieving 0.1 MPa accuracy. The arithmetic mean of the test results for six semi-prism specimens was taken as the measurement result. The ICCM specimens and the test fixtures for flexural and compressive strength (ICCM) are shown in Figure 6.

Shrinkage cracks were observed on the surface of the experimental ICCM samples (Figure 6a).

Analysis of the ICCM microstructure was performed on a Zeiss EVO MA 18 scanning electron microscope (Carl Zeiss Microscopy Ltd., Cambourne, UK) equipped with an X-Max 50N energy dispersive spectrometer (EDS) integrated into the electron microscope (Oxford Instruments, High Wycombe, UK). Key features are as follows: maximum microscope resolution—3 nm (30 kV, SE, W); accelerating voltage—0.2–30 kV; and magnification—5–1,000,000×. Detectors are as follows: SE—secondary electrons and HDBSD—backscattered electrons. The microscope allows for the examination of surface structure, phase, and chemical composition. For a complete examination of the sample, several preparatory steps must be performed. First, air must be evacuated from the chamber. Then, the accelerating voltage must be set, the operating modes of the equipment’s illumination system must be adjusted, and the electron probe must be adjusted so that it registers secondary electrons. The accelerating voltage must be adjusted to ensure optimal operation of the equipment.

One-way ANOVA included calculation of sum of squares, mean squares, degrees of freedom, and F- and *p*-values from ICCM property data with different clay contents (0%; 10%; 20%; 30%; 40%; 50%). The calculation result is considered statistically significant at *p* < 0.05 and *F* > 4.39. Critical *F* value (*p* ≤ 0.05) = 4.39. A statistical analysis was also performed using the Student’s *t*-test between the two groups—before and after the introduction of the MS additive. Group 1 comprised experimental ICCM compositions with the addition of clay (C); Group 2 comprised experimental ICCM compositions with the addition of clay (C) and microsilica (MS).

## 3. Results and Discussion

### 3.1. The Properties of Fresh ICCM

**Figure 7:**
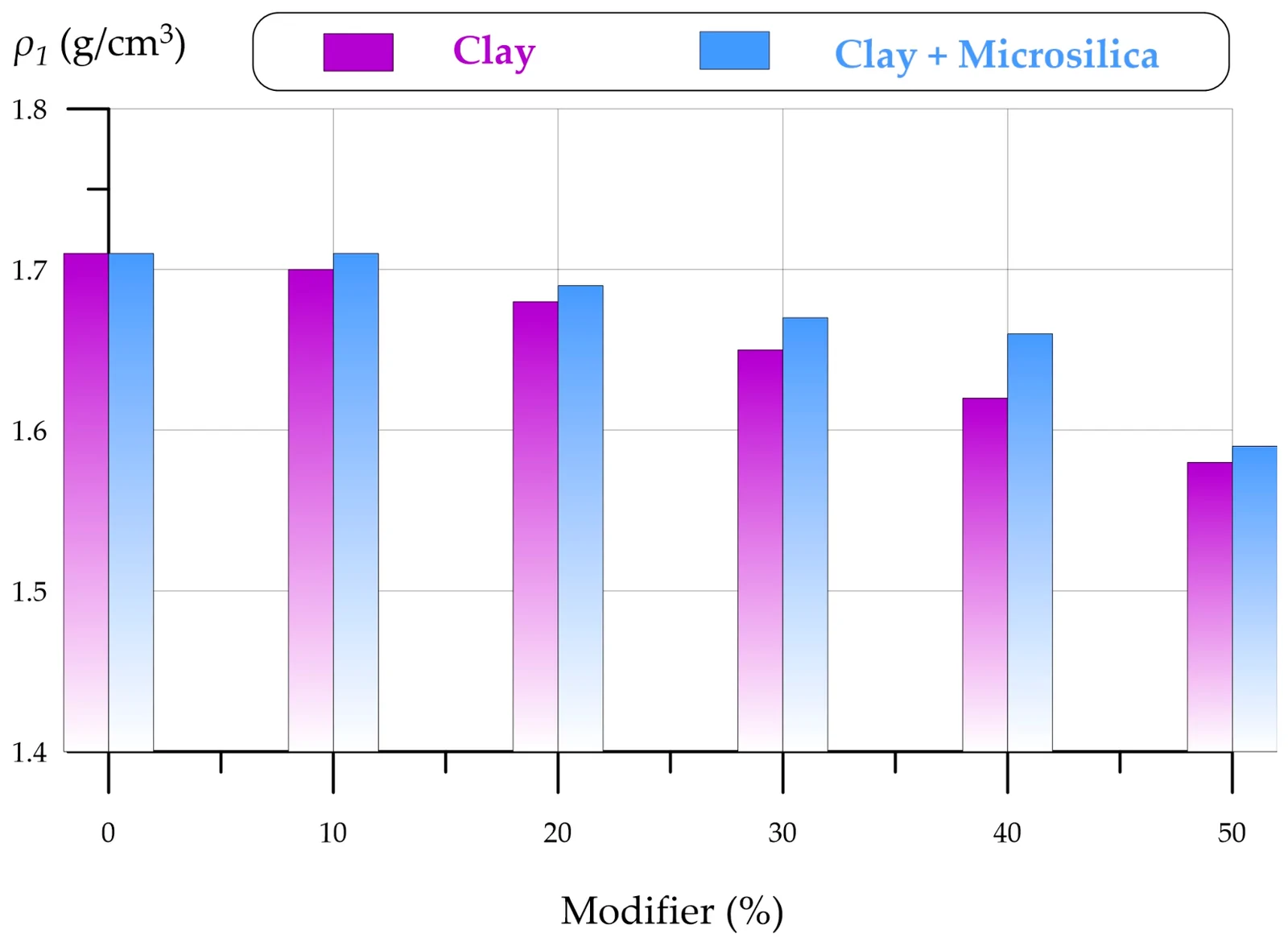
Density (*ρ*_1_) of fresh ICCM for soil stabilization as a function of C content and the presence of MS.

The density (*ρ*_1_) measurements for fresh ICCM featuring different clay percentages and comparable compositions with the addition of microsilica are shown in Figure 7.

**Figure 8:**
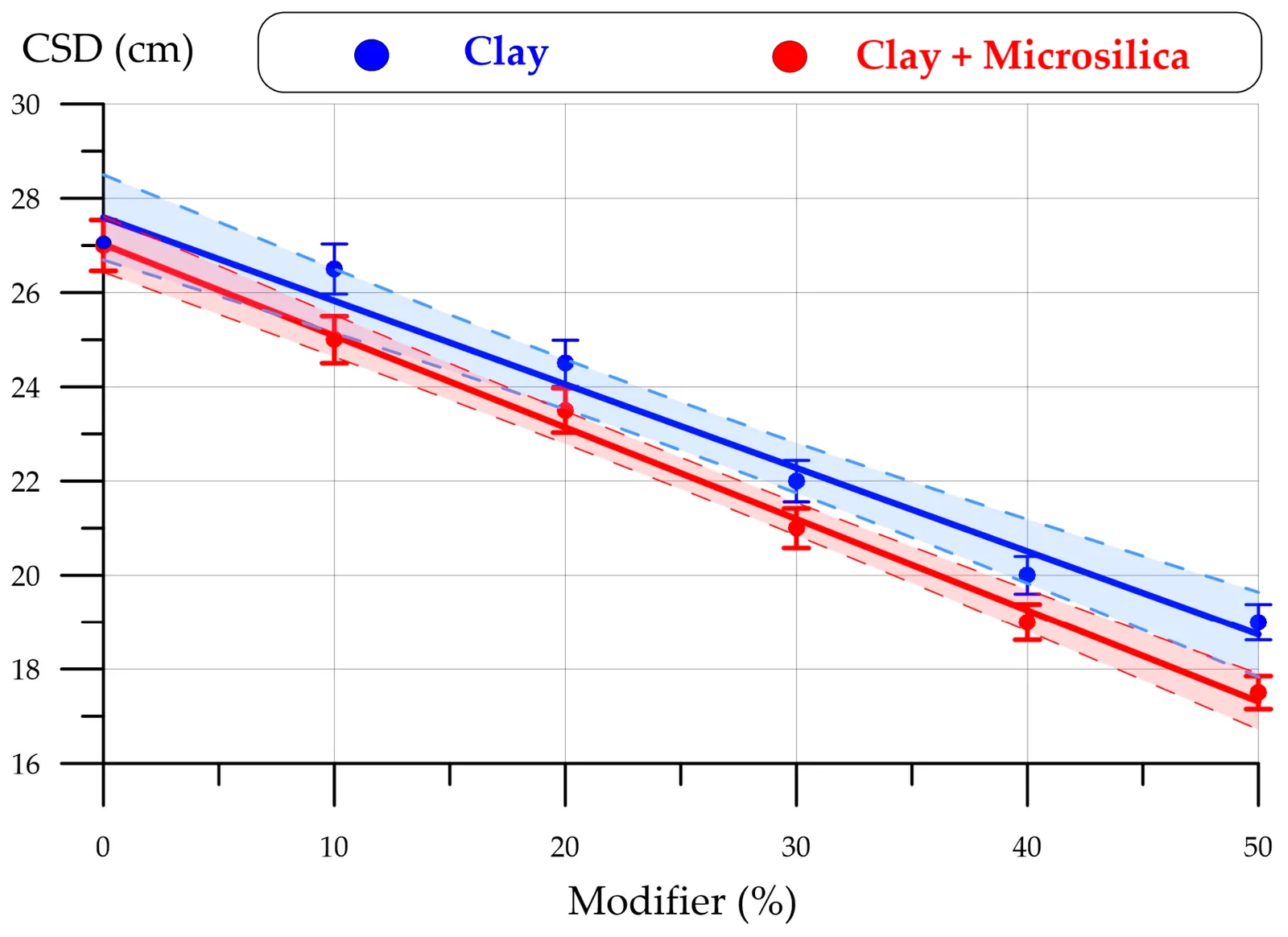
Dependence of the CSD of fresh ICCM for soil stabilization on the C content and the presence of MS.

The density of fresh ICCM for soil stabilization tends to decrease with increasing C content, which replaces part of the PC. The control 100PC composition has a density of 1.71 g/cm^3^. The density of the 50PC + 50C composition with a maximum C content of 50% is 1.58 g/cm^3^, which is 7.6% lower than that of the control specimen. The density of fresh ICCM modified with 2% MS also tends to decrease. The 50PC + 50C + 2MS composition has a density of 1.59 g/cm^3^, which is 7.0% lower than that of the control value. Fresh ICCM density drops as C content rises because clay particles are less dense. Clay particles also absorb water and increase the total volume of the liquid phase in the mixture, while the proportion of cement mortar decreases [26]. Figure 8 shows the graphical dependence of the cone spread diameter (CSD) of fresh ICCM on different clay contents and similar compositions additionally modified with MS.

**Figure 9:**
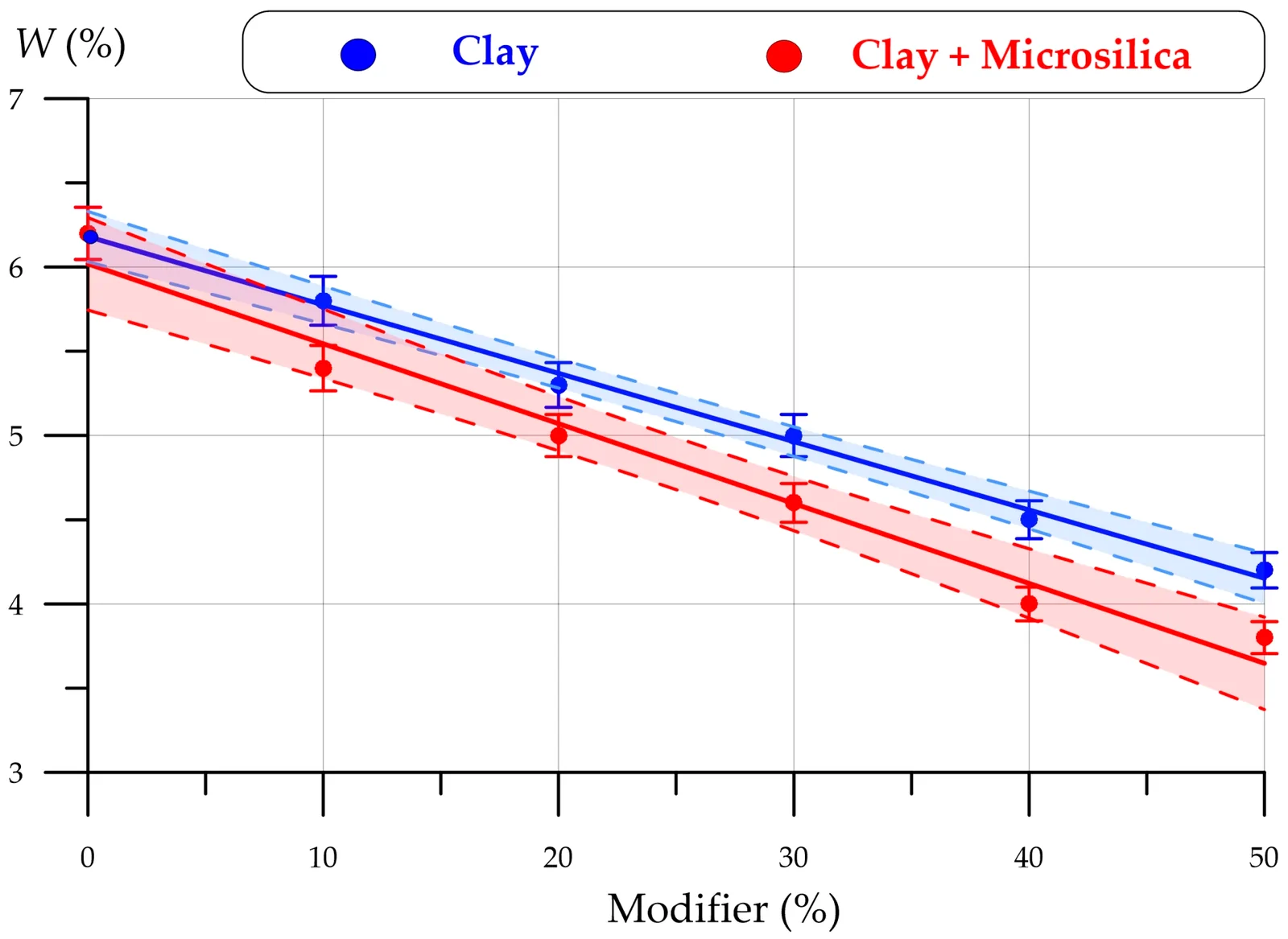
Water segregation (*W*) of fresh ICCM for soil stabilization as a function of C content and the presence of MS.

As seen in Figure 8, the cone spread diameter of fresh ICCM for soil stabilization decreases with increasing C content, which is added in place of part of the RS. The highest CSD value of 27.0 cm is characteristic of the control composition of the 100PC type. The CSD value for the 50PC + 50C composition was 19.0 cm, which is 29.6% lower than that of the control. A similar trend of decreasing CSD is observed for ICCM compositions modified with the addition of MS. When MS is included in ICCMs with different C contents from 0% to 50%, lower CSD values are recorded for all compositions. The 50PC + 50C + 2MS composition has a cone spread diameter of 17.5 cm, which is 35.2% less than that of the control composition. The reduction in CSD values with the addition of C and MS is associated with a number of effects. C and MS particles are highly dispersed; thus, a greater amount of water is required to wet them. Also, the presence of small MS particles increases the internal friction in the cement system, which leads to a decrease in the mobility of the entire system. As a result, the spreadability of the ICCM decreases, which is determined by smaller values of the cone spread diameter [46,47]. The dependences of the water segregation (*W*) of fresh ICCM on the amount of C and the presence of the MS modification are shown in Figure 9.

The water segregation of fresh ICCM for soil stabilization decreased from 6.2% for the control composition to 4.2% for the 50PC + 50C composition. The overall reduction in water segregation was 32.3%. ICCMs with different clay contents and modified MS exhibited lower water segregation values compared to compositions without MS. The water segregation of ICCM with the 50PC + 50C + 2MS composition was 3.8%, which is 38.7% less than that of the control specimen. The predominant mineral C is montmorillonite, which is characterized by high water-holding capacity. MS particles are characterized by high water demand. In addition to retaining water, C and MS particles fill the voids between cement particles, which hinders water migration to the upper layers of the fresh mortar and, as a result, reduces water segregation [26]. Table 7 displays the ANOVA results for the determination of the properties of fresh ICCM.

Statistically significant differences were observed between ICCM groups with different C contents, ranging from 0% to 50%. C content influences the soil stabilization properties of fresh ICCM. The results of statistical analysis using the Student’s *t*-test for independent samples are presented in Table 8. A comparison of the mean values was made between Group 1 (experimental ICCM compositions with the addition of clay (C)) and Group 2 (experimental ICCM compositions with the addition of clay (C) and microsilica (MS)).

Changes in the soil stabilization properties of fresh ICCM with C content as a percentage are presented in Table 9.

Experimental studies revealed the following characteristics of fresh injection mortars for soil stabilization:

– the density of fresh ICCM for stabilizing soils with a C content of 0% to 50% varies from 1.71 kg/m^3^ to 1.58 kg/m^3^. For compositions with 2% MS, the density ranges from 1.71 kg/m^3^ to 1.59 kg/m^3^;
– the cone spread diameter values of fresh ICCM for stabilizing soils with a C content of 0% to 50% vary from 27 cm to 19 cm. For compositions with 2% MS, the CSD values vary from 27 cm to 17.5 cm;
– the water separation of fresh ICCM for stabilizing soils with a C content of 0% to 50% varies from 6.2% to 4.2%, and for compositions with 2% MS, from 6.2% to 3.8%.

According to regulatory requirements [40], the density of injection mortars should be in the range from 1.1 to 2.2 g/cm^3^. The workability of the injection mortar mixture is regulated by the following series of workability grades: P1 with a CSD from 12 to 18 cm; P2 with a CSD from 18 to 24 cm; P3 with a CSD from 24 to 30 cm; and P4 with a CSD greater than 30 cm. Regarding water separation, injection mortars are divided into stable with water separation from 2% to 8%, conditionally stable with water separation from 8% to 16%, and unstable with water separation greater than 16% [38]. Thus, the developed ICCM compositions for soil stabilization, according to the properties of fresh ICCM, meet the regulatory requirements for density; have workability grades P1, P2 and P3; and are classified as stable solutions with respect to water separation.

### 3.2. The Properties of Hardened ICCM

**Figure 10:**
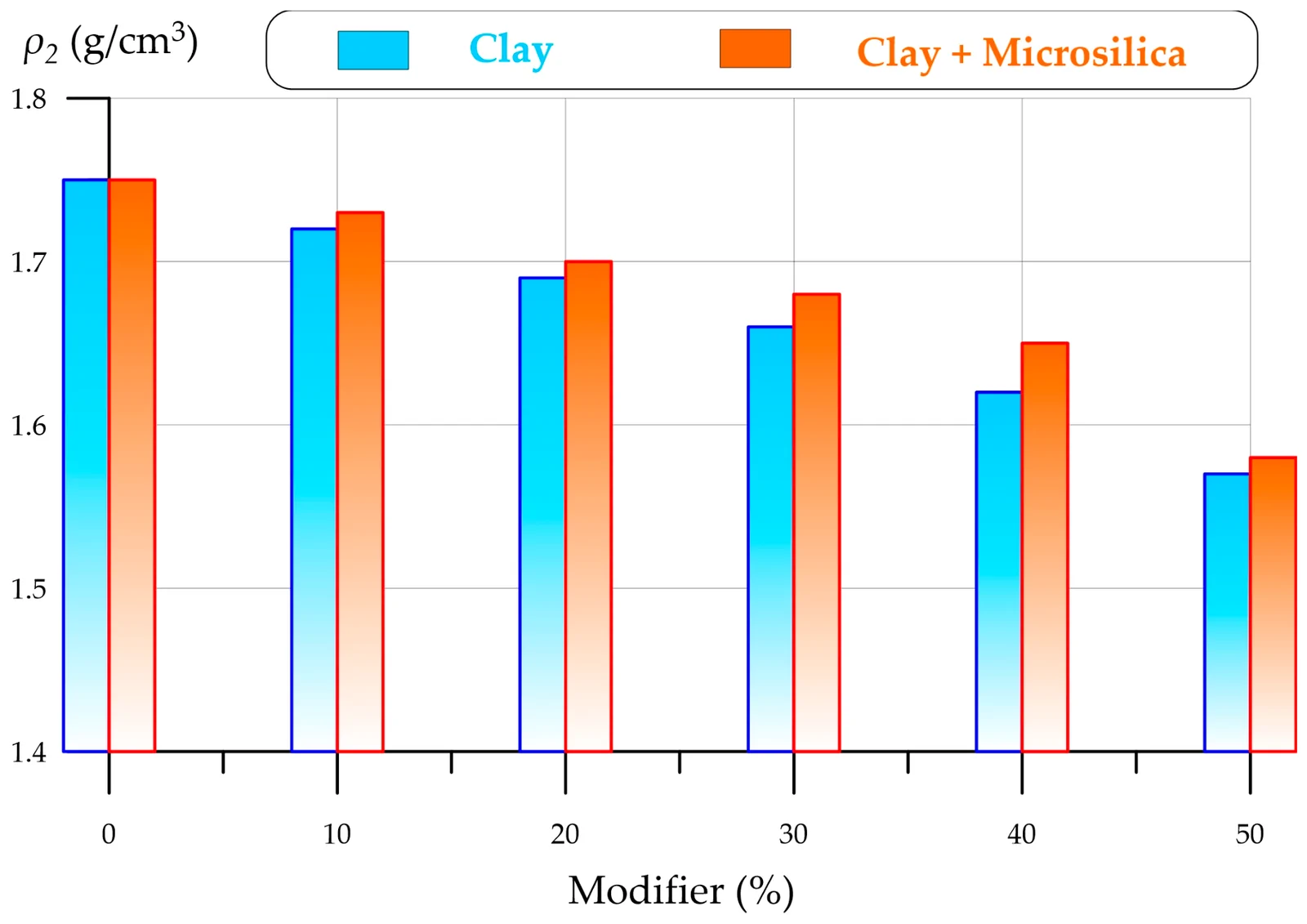
Relation of the density (*ρ*_2_) of ICCM for soil stabilization with the C content and the presence of MS.

**Figure 11:**
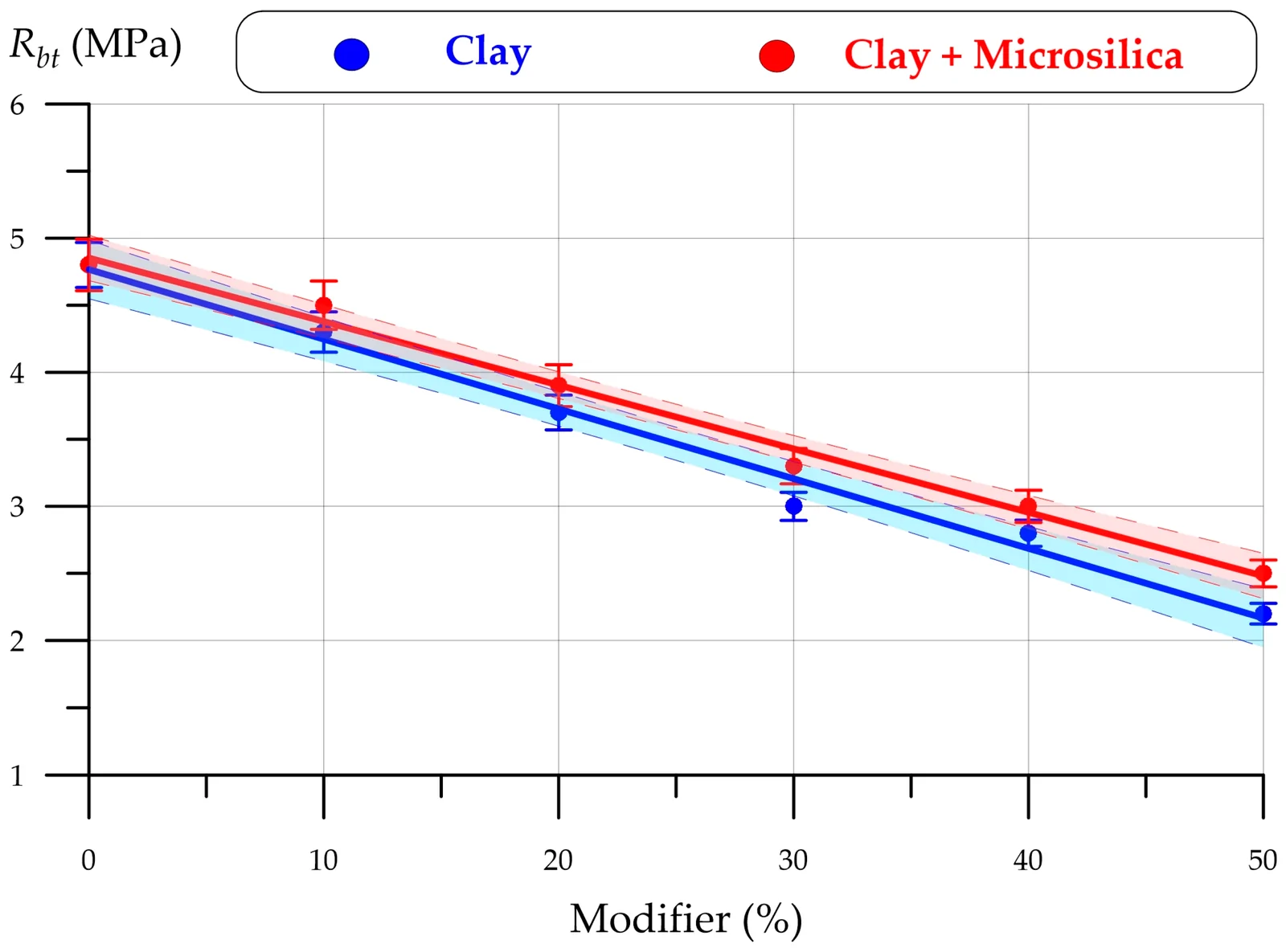
Flexural strength (*R_tb_*) of ICCM for soil stabilization versus C content and the presence of MS.

**Figure 12:**
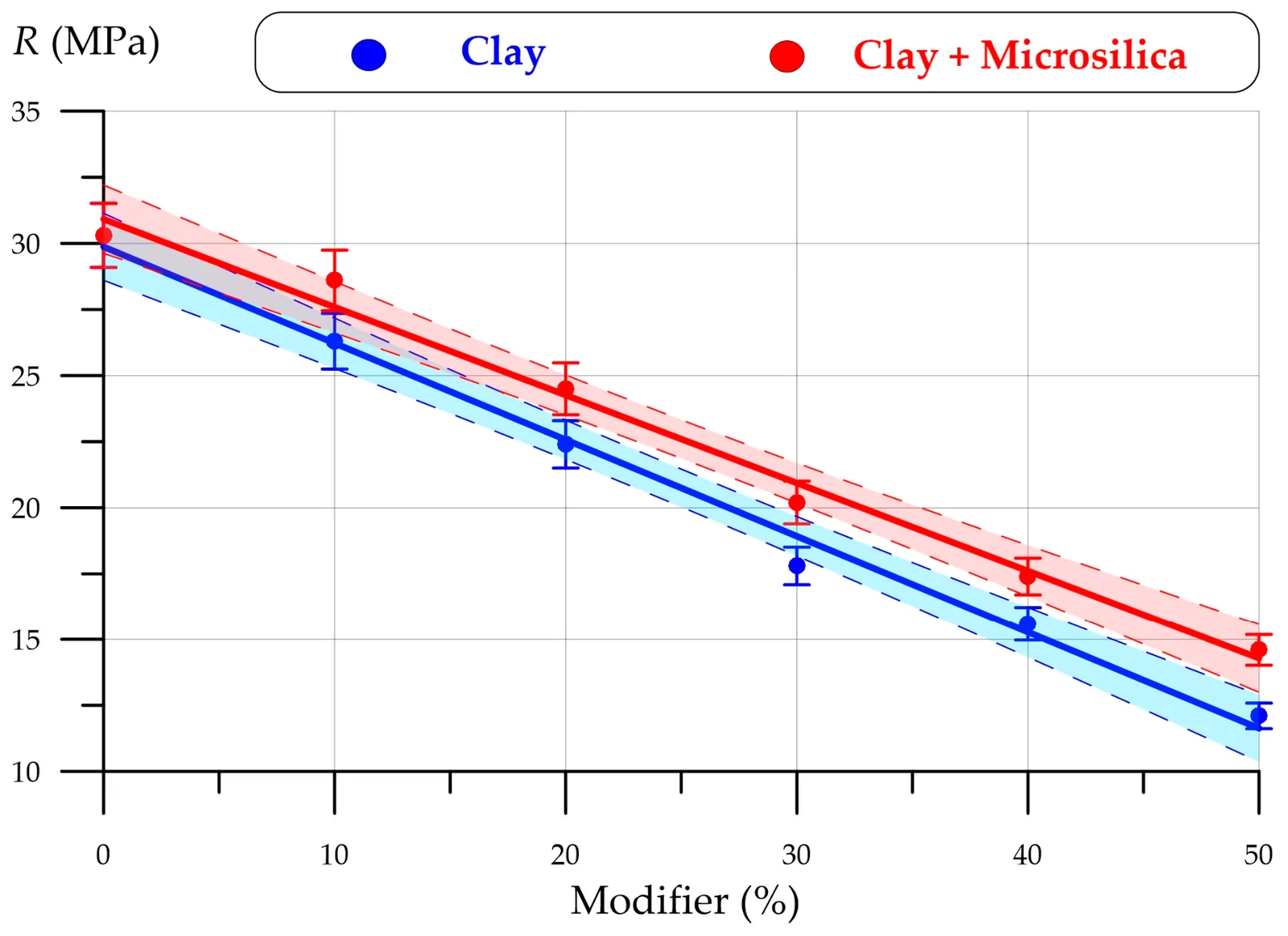
Compressive strength (*R*) of ICCM for soil stabilization versus C content and MS content.

The investigation focused on the density, flexural strength, and compressive strength of ICCMs with a clay content range of 0% to 50%. Similar ICCMs additionally modified with 2% MS are presented in Figure 10, Figure 11 and Figure 12. The relationship between hardened ICCM density (*ρ*_2_), clay content, and microsilica presence is depicted in Figure 10.

The density of a hardened ICCM for soil stabilization, similarly to fresh mortars, decreases as C content rises. The control 100PC mortar exhibits a density measuring 1.75 g/cm^3^. The 50PC + 50C mortar has a density of 1.57 g/cm^3^, which is 10.3% lower than that of the control value. The density of ICCM mortars with 2% MS also decreased with increasing C content. However, compared to mortars without MS, the rate of density reduction was slightly less. The 50PC + 50C + 2MS mortar had a density of 1.58 g/cm^3^, which is 9.7% smaller than that of the control composition. The decrease in ICCM density with the inclusion of C occurs because the density of PC particles is higher than the density of C particles. Consequently, substituting clay for a portion of the cement reduces the solid phase’s overall density, leading to a decrease in the mixture’s density. The flexural strength (*R*_tb_) of an ICCM, as depicted in Figure 11, is shown to depend on both the clay content and the inclusion of microsilica.

Figure 11 illustrates that adding more C instead of PC weakens the flexural strength of ICCMs for soil stabilization. For the control composition, the highest flexural strength reached was 4.8 MPa. For the 50PC + 50C composition, which had 50% C, the lowest flexural strength was observed. The decrease in flexural strength was 54.2%. ICCMs for soil stabilization modified with MS had higher flexural strength values than compositions without MS. For example, the 50PC + 50C + 2MS composition exhibited a flexural strength of 2.5 MPa, which is 47.9% lower than that of the control. Figure 12 illustrates how the compressive strength (*R*) of ICCM changes with the amount of clay and the inclusion of microsilica.

As C content rises from 0% to 50%, the compressive strength of ICCMs used for soil stabilization declines. The control composition achieved a maximum compressive strength of 30.3 MPa. For the 50PC + 50C mix, the lowest compressive strength observed was 12.1 MPa, which represents a 60.1% decrease compared to the control. The 50PC + 50C + 2MS composition exhibits a compressive strength of 14.6 MPa, which represents a 51.8% decrease compared to the control.

The reduction in the strength properties of ICCMs for soil stabilization when part of the Portland cement is replaced with clay is explained by the following mechanisms. The amount of the main binder component decreases, and, as a result, the total amount of calcium hydrosilicates (C-S-H) and calcium hydroaluminates (C-A-H), which are responsible for the formation of the cement matrix and its strength, decreases. C particles envelop the PC particles, which complicates the hydration process of some PC grains [26,48]. In addition, the structure of the cement–clay matrix is disrupted, and weak zones appear, as a result of which the ICCM loses strength. The inclusion of an additional highly dispersed amorphous additive MS in the cement–clay matrix has a positive effect on its strength properties. MS is characterized by high reactivity, actively interacts with free calcium hydroxide Ca(OH)_2_ and promotes the growth of additional C-S-H gels. The transition zone between C and PC particles is strengthened, the total number of capillary pores is reduced, and the matrix structure becomes more uniform and durable [49].

The analysis of variance (ANOVA) for the hardened ICCM property determination is shown in Table 10.

Statistically significant differences were observed between ICCM groups with different C contents ranging from 0% to 50%. C content affects the properties of hardened ICCMs for soil stabilization. Table 11 presents the results of the statistical analysis using the Student’s *t*-test between Group 1 for the experimental ICCM compositions with the addition of clay (C) and Group 2 for the experimental ICCM compositions with the addition of clay (C) and microsilica (MS).

Statistically significant differences were observed between the ICCM groups with and without MS addition. MS content affects the soil stabilization properties of hardened ICCM.

Table 12 contains the percentage changes regarding the properties of hardened ICCMs used for soil stabilization.

The following findings were determined from the experimental studies:

– the density of ICCMs for stabilizing soils with a C content of 0% to 50% varies from 1.75 kg/m^3^ to 1.57 kg/m^3^. For compositions with 2% MS, the density varies from 1.75 kg/m^3^ to 1.58 kg/m^3^;
– the flexural strength of ICCMs for stabilizing soils with a C content of 0% to 50% varies from 4.8 MPa to 2.2 MPa, and for compositions with 2% MS, the flexural strength varies from 4.8 MPa to 2.5 MPa;
– the compressive strength of ICCMs for soil stabilization with a C content of 0% to 50% varies from 30.3 MPa to 12.1 MPa, while for compositions with 2% MS, the compressive strength varies from 30.3 MPa to 14.6 MPa.

According to regulatory requirements [38], the compressive strength of hardened cement-based injection mortars must be at least 5 MPa. Thus, the ICCMs developed in this study for soil stabilization comply with regulatory requirements and can be considered a more cost-effective and environmentally friendly alternative to injection mortars made using cement alone. It is worth noting that ICCM compositions modified with MS additive are more effective than similar compositions without the additive. They are characterized by lower water segregation values and higher strength properties. Compositions of the following types are the most optimal for use in real construction practice: 90PC + 10C + 2MS, 80PC + 20C + 2MS, 70PC + 30C + 2MS, 60PC + 40C + 2MS and 50PC + 50C + 2MS.

### 3.3. Results of SEM Analysis of the ICCM Structure

**Figure 13:**
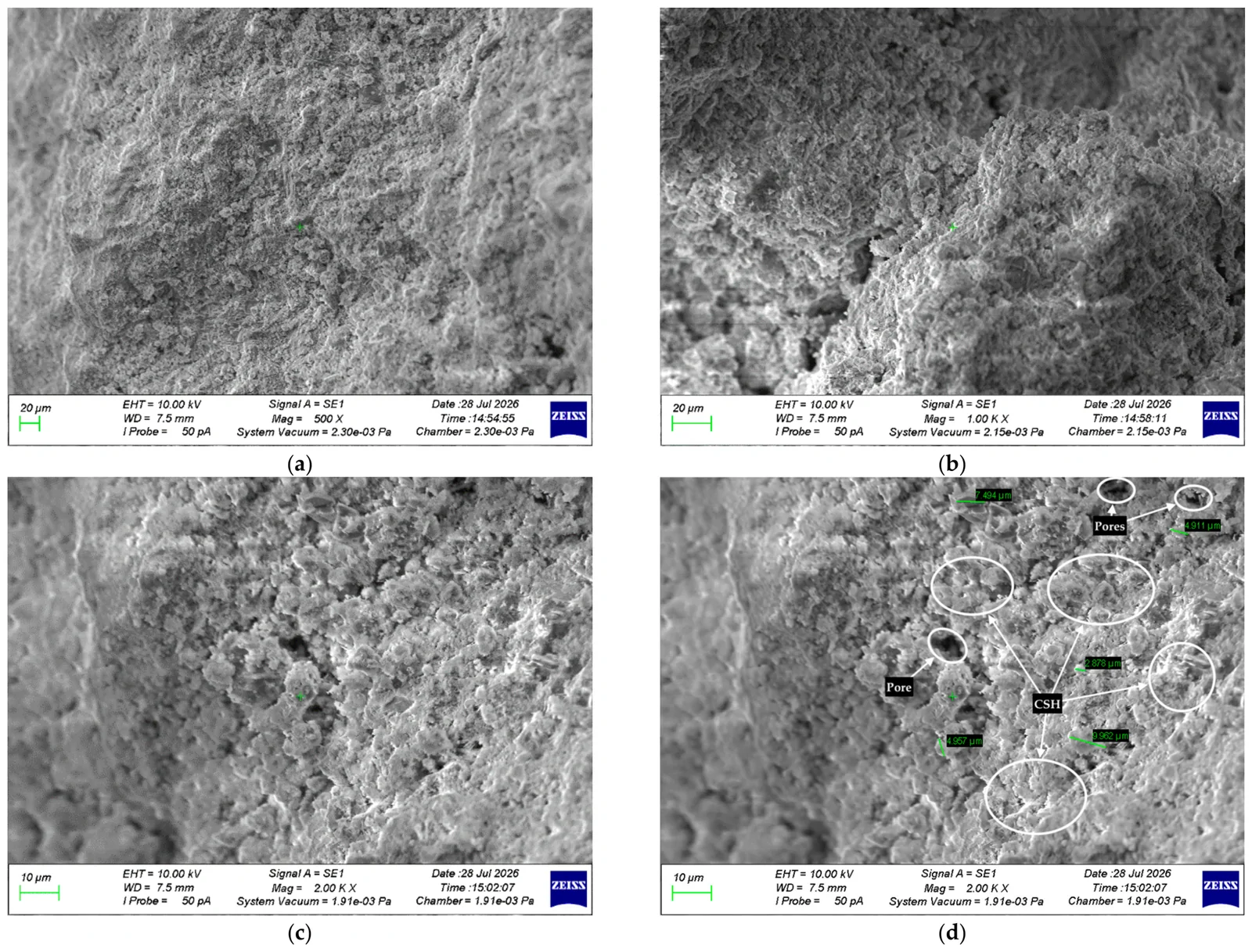

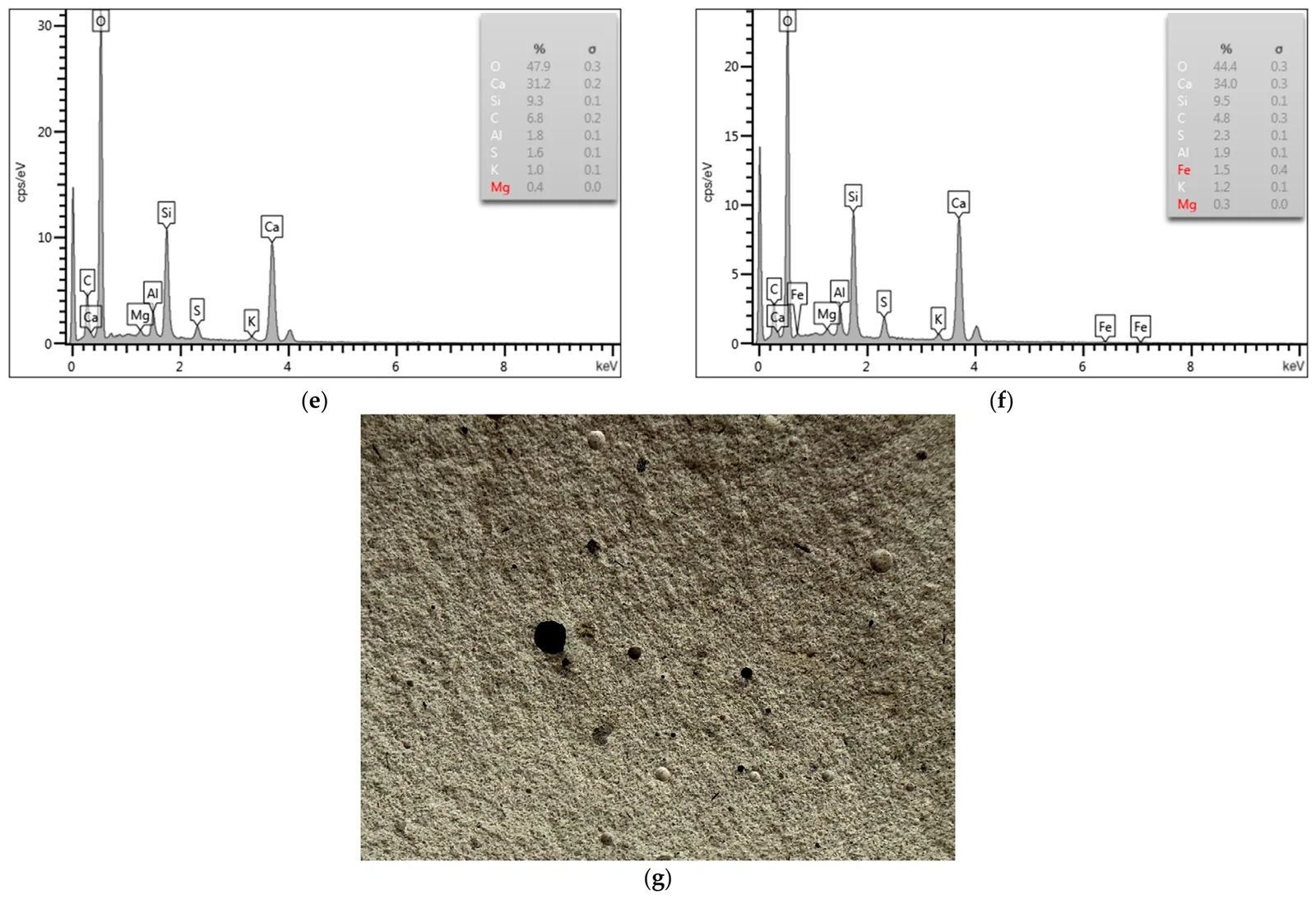
SEM images of the structure and EDS of the ICCM of the 100PC composition at magnifications of (**a**) 500×, (**b**) 1000×, (**c**) 2000×, and (**d**) 2000× (with markings); (**e**) spectrum 1; (**f**) spectrum 2; (**g**) fracture.

**Figure 14:**
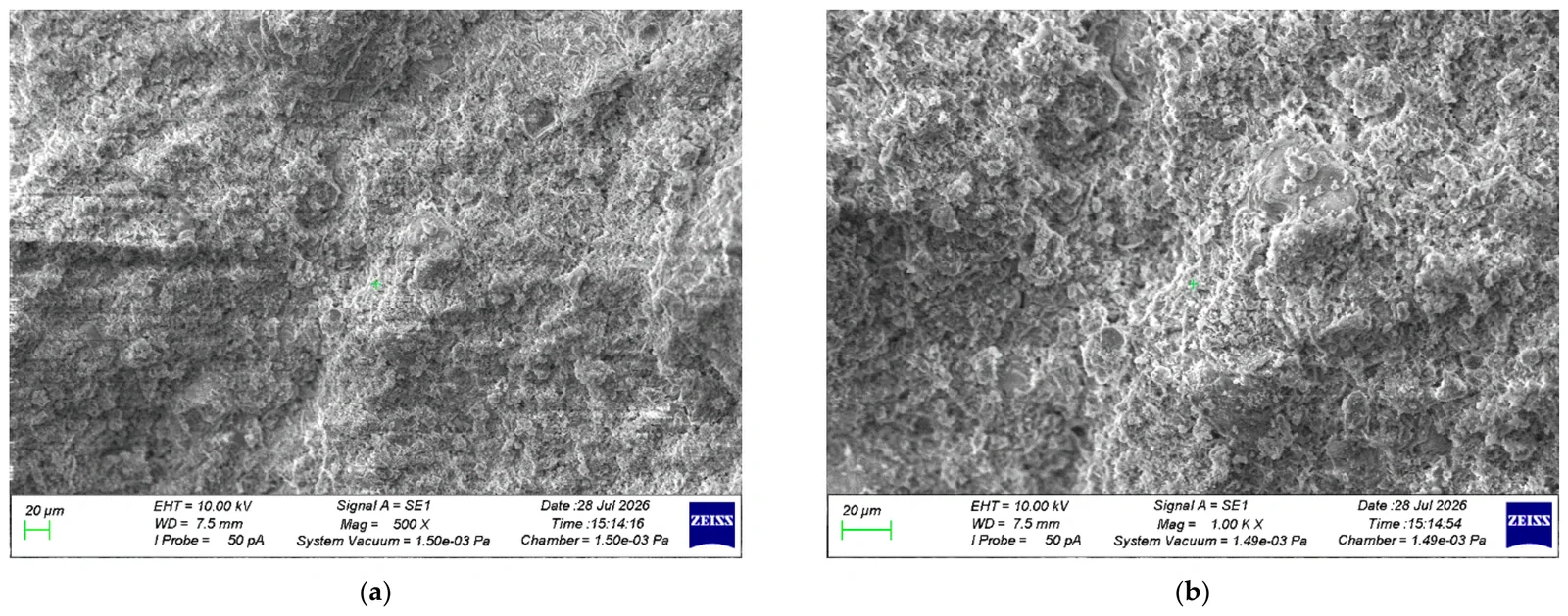

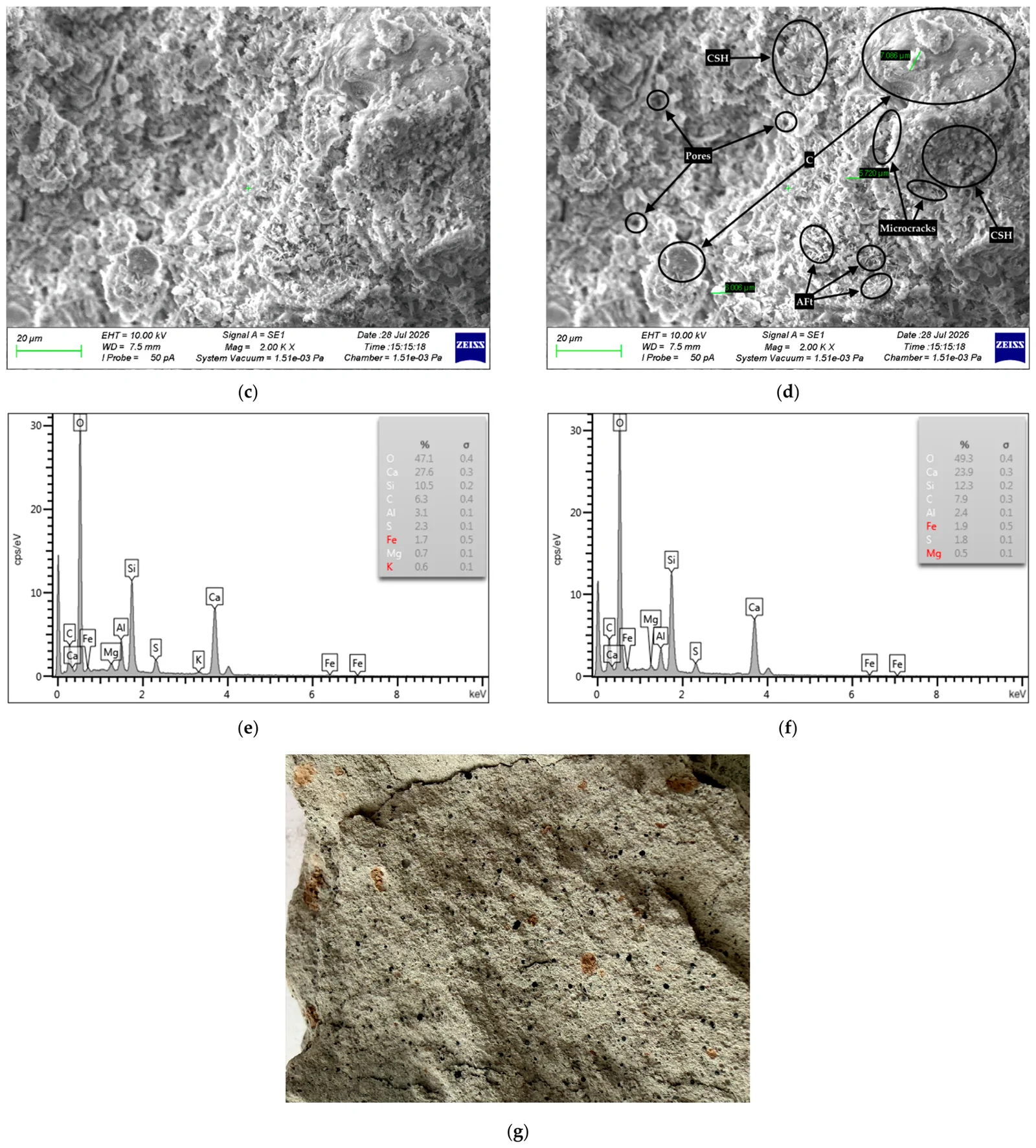
SEM images of the ICCM structure of the 70PC + 30C + 2MS composition at magnifications of (**a**) 500×, (**b**) 1000×, (**c**) 2000×, and (**d**) 2000× (with markings); (**e**) spectrum 1; (**f**) spectrum 2; (**g**) fracture.

**Figure 15:**
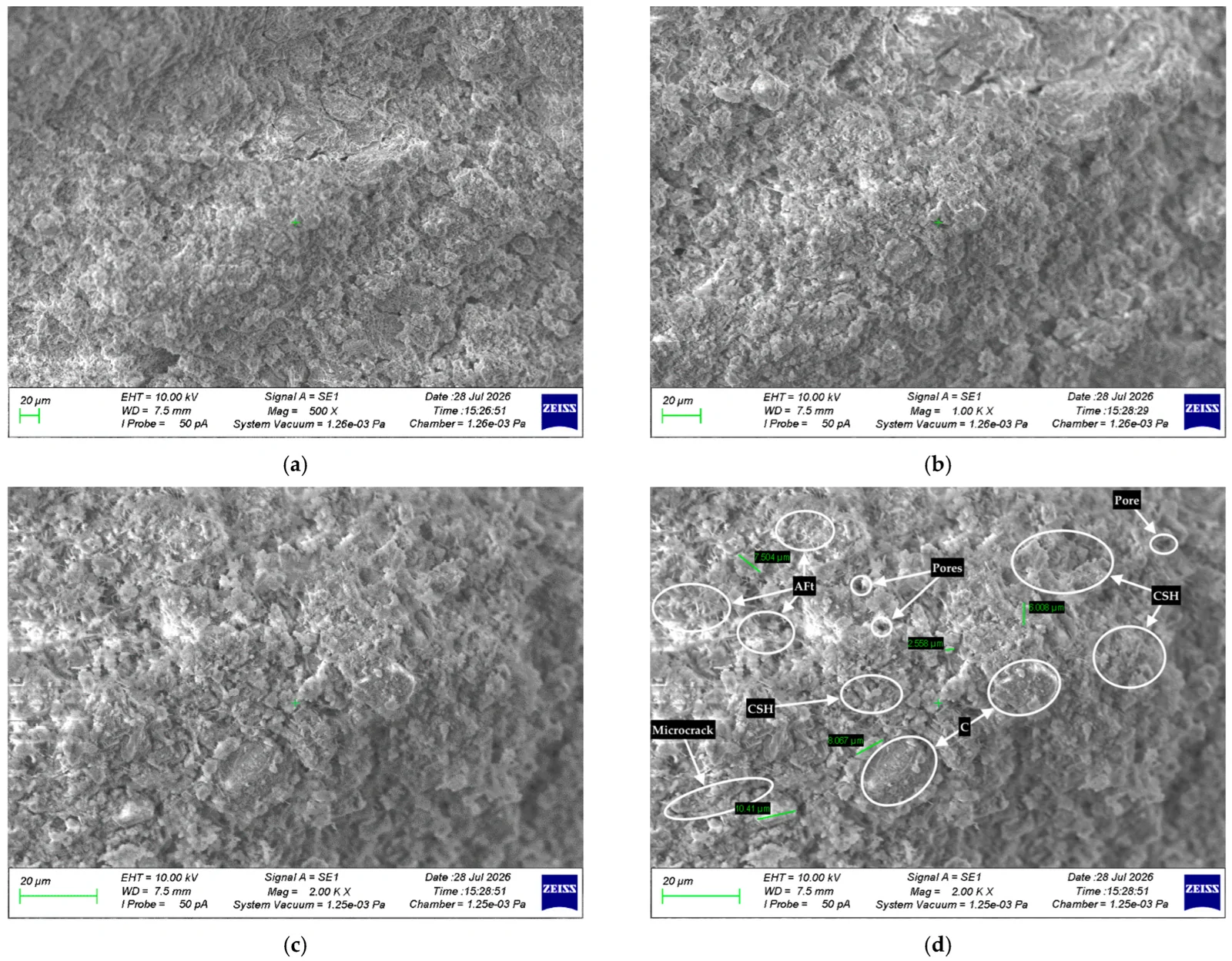

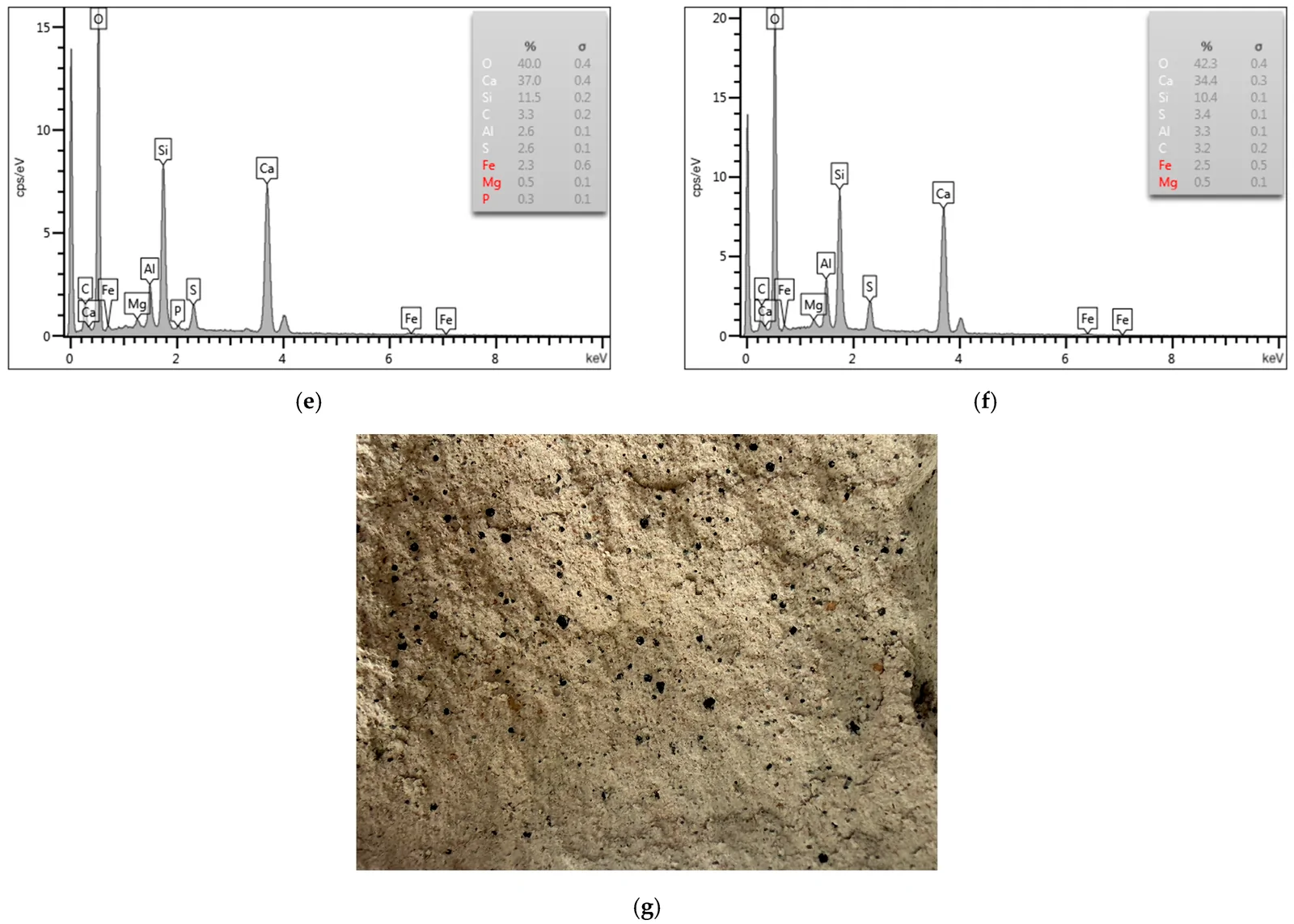
SEM images of the ICCM structure of the 50PC + 50C + 2MS composition at magnifications of (**a**) 500×, (**b**) 1000×, (**c**) 2000×, and (**d**) 2000× (with markings); (**e**) spectrum 1; (**f**) spectrum 2; (**g**) fracture.

A comparative analysis of the morphological features, microstructure and macrostructure of ICCMs for soil stabilization was performed for the 100PC control composition and the 70PC + 30C + 2MS and 50PC + 50C + 2MS compositions. Figure 13, Figure 14 and Figure 15 show microstructure images of these compositions at various magnifications. Figure 13 shows photographs of the structure and energy diffraction X-ray patterns of the ICCM control composition (without clay or microsilica).

The matrix of the injection solution of the control composition is homogeneous. A small number of pores is present. Clusters of hydration reaction products are observed: C-S-H and C-A-H. The microstructure image (Figure 14b) shows microcracks. The EDS spectra reveal regions with high Ca and Si contents, which may correspond to the C-S-H phase. The images of the fracture macrostructure show pores and a developed network of cracks. Figure 14 shows photographs of the structure and energy diffraction X-ray patterns of the ICCM of the 70PC + 30C + 2MS composition.

The injection cement–clay mortar of the 70PC + 30C + 2MS composition has a homogeneous structure with pores and a network of microcracks. Agglomerated clusters of clay particles and products of the C-S-H and C-A-H hydration reactions are observed. Clusters of ettringite crystal zones (AFt) are observed. Microcracks are concentrated in the phase interface zones between the cement matrix and agglomerated clusters of clay particles. The EDS spectra also reveal areas with high Ca and Si contents, which may correspond to the Ca(OH)_2_ and C-S-H phases. The images of the fracture macrostructure clearly show pores, a developed network of cracks, and agglomerated clusters of C and MS. Figure 15 shows the structure and EDS images of the ICCM of the 50PC + 50C + 2MS composition.

Injectable cement–clay mortars of the 50PC + 50C + 2MS type are also characterized by a homogeneous matrix with the presence of pores, microcracks, and a larger number of agglomerated clusters of clay particles compared to the 70PC + 30C + 2MS type composition. Zones of C-S-H accumulation and ettringite crystals (AFt) are observed. The EDS spectra reveal regions with high Ca and Si contents, which may correspond to the CSH phase. Images of the fracture macrostructure also reveal pores, a developed crack network, and agglomerated clusters of C and MS.

### 3.4. ICCM Carbon Emissions Calculation Results

To identify the environmental benefits of the developed solutions, the carbon emissions of 2% MS injection cement–clay slurries for soil stabilization per cubic meter were calculated (Table 13).

Based on the calculation of carbon emissions associated with the production of 1 m^3^ of ICCM for soil stabilization, it was found that as the proportion of clay replacing part of the Portland cement increases by 10%, 20%, 30%, 40% and 50%, the total amount of carbon emissions decreases by 8.4%, 18.3%, 28.1%, 37.9% and 47.8%, respectively.

### 3.5. Discussion

Other researchers have also assessed the applicability of clays for the production of cementitious composites for various purposes (Table 14), and numerous experimental studies have confirmed the feasibility of using clay for the production of environmentally friendly composites with reduced cement content.

**Figure 16:**
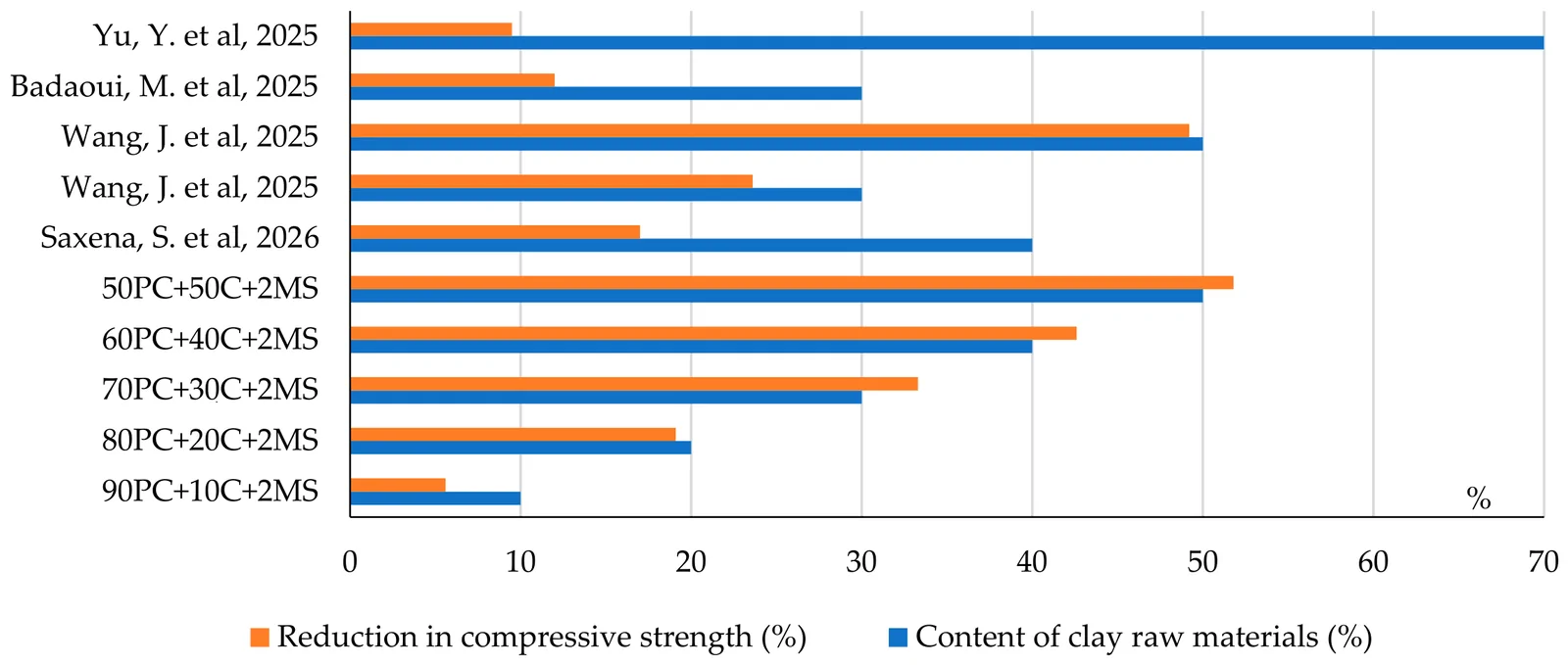
Comparative diagram illustrating the decrease in composite strength with a certain clay content [57,59,61,62].

The results of the evaluation of the properties of cement–clay composites presented in this study and the analyzed works of other authors [51,52,53,54,55,56,57,58,59] confirm the promise and rationality of using clay raw materials in cement composites as a material to replace part of the Portland cement. Effective cement–clay mortars for the purposes of injection into water-bearing sandy soils have already been developed previously [26]. As in the present work, in Ref. [26], a decrease in the fluidity of the solutions and a decrease in their water separation with an increase in the clay content were recorded. In the work of Ref. [59], the introduction of silty clay powder instead of part of the Portland cement up to 50% significantly reduced the fluidity of the solutions and their strength properties, which is consistent with the results obtained in the present work. The introduction of clay in optimal quantities makes it possible to obtain composites with the required performance properties for specific practical problems, one of which is the development of more environmentally friendly and cost-effective solutions for soil stabilization. However, both in this study and in the works of other authors, when replacing part of the cement with clay raw materials, a decrease in strength properties is recorded [59,60,61,62]. Figure 16 shows a comparative diagram showing the proportion of clay raw materials in cement composites and the reduction in compressive strength.

Thus, it is evident from Figure 16 that when replacing cement with clay raw materials by 10% to 70%, the reduction in compressive strength amounts to 5.6–51.8%, respectively. It is important to take into account the individual characteristics of the clay raw material, namely, its mineralogical and granulometric composition, the method of preparing the raw material before introducing it into the cement composite (drying, firing, additional grinding), the water–binder ratio of the mixture and the proportion of clay raw material replacing part of the binder. The results obtained in this study demonstrate a clear trend toward a decrease in compressive strength with an increase in the content of clay raw materials. In Refs. [61,62], clay raw materials were subjected to additional firing, which increased their effectiveness. In general, based on the results of the microstructural and macrostructural analysis of ICCMs for soil stabilization, the following general conclusions can be drawn. All the studied compositions are characterized by the presence of microcracks, pores, and agglomerated clusters of C and MS in the structure of the cement matrix. The matrix structure itself is quite homogeneous. When developing cement–clay composites, in addition to the positive effects associated with reduced Portland cement consumption and a reduced carbon footprint, it is important to consider potential risks. Firstly, this is a significant reduction in the mobility of the mortars due to the high water demand of the clay particles and, consequently, a decrease in their strength properties. Secondly, clay is an unstable raw material, and, depending on the deposit, its mineralogical composition and other properties can vary significantly. This will require a series of additional experimental studies on the development of injection cement–clay mortars for soil stabilization, taking into account the properties of the specific clay raw material and the individual geological conditions of the site (soil type, soil water saturation, temperature conditions) [26,61,62,63]. Variations in the mineral composition of clay can significantly affect the soil stabilization properties of ICCMs. Given the variations in montmorillonite and kaolinite content in clay raw materials depending on the deposit, the following formulation adjustments can be applied. For clays with a high montmorillonite content, whose particles actively swell in water, the overall proportion of clay in the solution can be reduced and special stabilizing plasticizing additives can be added to prevent segregation. For clays with a predominance of kaolinite, an increase in the proportion of clay in the solution is permissible with the use of special additives that regulate setting time. Before the practical use of injection cement–clay mortars for soil stabilization, it is necessary to conduct laboratory tests on local soils and adapt the mortar formulations to the specific geotechnical conditions of the region.

In this study, injection cement–clay mortars were developed for soil consolidation with up to 50% Portland cement savings, up to 47.8% reduced carbon emissions and optimal performance properties. This work does not cover the durability aspects of ICCMs for soil consolidation, such as resistance to chloride ion penetration and frost resistance. These properties are essential for long service life. Directions for further research will focus on a thorough study of the durability properties of cement–clay mortars for soil consolidation. The overall contribution to sustainable development is achieved by reducing the carbon footprint, ensuring the rational use of resources, and optimizing properties for a specific practical task. Some of the cement is replaced by clay, and the total amount of carbon emissions associated with the production of 1 m^3^ of ICCM for soil consolidation is reduced.

## 4. Conclusions

This study proposes a new cement–clay-based material for the injection stabilization of soft and unstable soils. The properties of fresh and hardened injection cement–clay mortars (ICCMs) for stabilizing soils with varying clay (C) contents from 0% to 50%, modified with microsilica (MS), were evaluated. Comparative SEM and EDS analyses of ICCMs with varying C contents and modified MS were performed.

(1) ICCM is a specialized mortar designed primarily for the stabilization of water-bearing sandy soils, cohesive soils, and fractured rocky soils. This material consists of cement, clay, and microsilica additives, which demonstrate effective interactions. The inclusion of clay in place of a portion of the cement reduces the density of ICCMs and increases their stability. The MS additive promotes the active growth of additional C-S-H gels and improves the strength of ICCMs for stabilizing soils with different C contents.
(2) The density of fresh ICCM for stabilizing soils with C contents from 0% to 50% decreases with increasing C: for compositions with a maximum clay content of 50%, the density decrease was 7.6%, and for a composition with 2% MS, it was 7.0%. The mobility of the ICCM, as determined by the CSD value, decreases with an increase in clay content from 0% to 50%. Thus, for the ICCM with 50% clay, the CSD value decreased by 29.6%, and for the composition with the MS additive, by 35.2%. The water segregation of the ICCMs decreased with increasing C content. The water segregation for the mixture with 50% clay decreased by 32.3%, and for the mixture with the MS additive, by 38.7%.
(3) The density of a hardened ICCM for soil stabilization decreases with increasing C content. For the ICCM with 50% C, the density decrease was 10.3%, while for the composition with 2% MS, it was 9.7%. The flexural and compressive strength of an ICCM decreases with increasing C. For the composition with 50% C, the decreases in flexural and compressive strength were 54.2% and 60.1%, respectively. For the composition with 50% C and 2% MS, the decreases in flexural and compressive strength were 47.9% and 51.8%, respectively.
(4) The optimal ratios of raw components for the production of ICCMs for soil stabilization were determined: a water–solid ratio of 0.6; Portland cement content from 90% to 50%; clay content from 10% to 50%; and a microsilica content of 2% of the weight of Portland cement and clay.
(5) The microstructure of the ICCM for soil stabilization is homogeneous. Pores, microcracks, agglomerated clay clusters, and accumulations of C-S-H, C-A-H, and AFt hydration reaction products are observed.
(6) The developed ICCMs with MS for soil stabilization exhibit optimal performance properties and have potential for application in real-world construction. However, when implementing these studies, it is important to consider the limitations of this study, namely, the lack of durability testing, the fixed water-to-binder ratio of 0.6, and the use of the same clay raw material.
(7) This study contributes to sustainable development by reducing CO_2_ emissions and improving the rational use of raw materials. Replacing Portland cement, which accounts for the majority of carbon emissions, with environmentally friendly clay raw materials reduces the carbon emissions associated with the production of 1 m^3^ of ICCM by 8.4%, 18.3%, 28.1%, 37.9%, and 47.8% when replacing 10%, 20%, 30%, 40%, and 50%, respectively. It is important to note that the carbon footprint reduction calculation performed relates to the specific ICCMs for soil stabilization with MS presented in this study and is for informational purposes only. In the case of actual practical application, the calculation data may be adjusted.

## Figures and Tables

**Table 1:** Properties of ICCMs.

**SiO_2_ (%)**	**Al_2_O_3_ (%)**	**Fe_2_O_3_ (%)**	**CaO (%)**	**MgO (%)**	**Na_2_O (%)**	**SO_3_ (%)**	**Cl^−^(%)**	**LOI (%)**
18.5	5.3	4.8	65.8	1.7	0.4	2.7	0.003	0.797
**Characteristics**				**Actual value**				
Blaine Specific Surface Area (cm^2^/g)				3300				
Normal Density (%)				29.5				
Setting Time (min)				200				
Setting Time (min)				310				
Compressive Strength (MPa)				58.6				
Bending Strength (MPa)				8.2				

**Table 2:** MS Properties.

**SiO_2_ (%)**	**Al_2_O_3_ (%)**	**Fe_2_O_3_ (%)**	**CaO (%)**	**MgO (%)**	**Na_2_O (%)**	**K_2_O (%)**	**C (%)**	**LOI (%)**
87.1	0.66	0.85	1.5	1.03	0.61	1.23	5.94	1.08
**Characteristics**				**Actual value**				
Bulk density (kg/m^3^)				155				

**Table 3:** Properties of C.

**LOI (%)**	**SiO_2_ (%)**	**Al_2_O_3_ (%)**	**Fe_2_O_3_ (%)**	**CaO (%)**	**MgO (%)**	**SO_3_ (%)**	**K_2_O (%)**	**Na_2_O (%)**	**P_2_O_5_ (%)**	**TiO_2_ (%)**
13.44	47.92	14.85	6.38	10.51	3.15	0.14	2.19	0.66	0.02	0.74
**Characteristics**					**Actual value**					
Density (g/cm^3^)					2.46					
Yield point (%)					41.7					
Plasticity limit (%)					21.2					
Plasticity index					20.5					

**Table 4:** Clay particle size distribution.

Content of Fractions (µm), % by Weight					
>63	50–63	10–50	5–10	1–5	Less than 1
1.45	0.27	2.94	17.83	17.06	60.45

**Table 5:** Experimental ICCM Compositions.

Composition Type	PC (%)	C (%)	MS (%)
100PC	100	0	0
90PC + 10C	90	10	0
80PC + 20C	80	20	0
70PC + 30C	70	30	0
60PC + 40C	60	40	0
50PC + 50C	50	50	0
90PC + 10C + 2MS	90	10	2
80PC + 20C + 2MS	80	20	2
70PC + 30C + 2MS	70	30	2
60PC + 40C + 2MS	60	40	2
50PC + 50C + 2MS	50	50	2

**Table 6:** Calculation formulas.

Number	Formula	Description
(1)	$\rho_{1}=\frac{m_{2}-m_{1}}{V}$	$\rho_{1}$—density of fresh ICCM (g/cm^3^); *m*_1_—mass of an empty pycnometer (g); *m*_2_—mass of the pycnometer with cement paste (g); *V*—pycnometer capacity (cm^3^)
(2)	$W=\frac{a-b}{a}$	*W*—water separation of fresh ICCM; *a*—initial volume of ICCM (cm^3^); *b*—volume of settled ICCM (cm^3^)
(3)	$\rho_{2}=\frac{m}{V}$	$\rho_{2}$—density of the hardened ICCM (g/cm^3^); *m*—specimen mass (g); *V*—specimen volume (cm^3^).
(4)	$R=\frac{F}{S}$	*R*—compressive strength of ICCM (MPa); *F*—failure force (N); *S*—pressure plate area (2500 mm^2^)
(5)	$R_{tb}=\frac{1.5\cdot F\cdot l}{b^{3}}$	*R_tb_*—flexural strength of hardened ICCM (MPa); *F*—failure force (N); *l*—distance between support axes (mm); *b*—side of the square section of the prism sample (mm)

**Table 7:** Analysis of variance for the results of determining the properties of fresh ICCM.

	Degrees of Freedom DF	Sum of Squares SS	Mean Square MS	F-Stat	*p*-Value	Conclusion
$\rho_{1}$						
Between Groups	5	0.0221	0.0044	23.0857	0.0007	Significant
Within Groups	6	0.0012	0.0002			
Total:	11	0.0233				
CSD						
Between Groups	5	121.9167	24.3833	39.0119	0.0002	Significant
Within Groups	6	3.7501	0.625			
Total:	11	125.6668				
*W*						
Between Groups	5	6.85	1.37	20.0514	0.0011	Significant
Within Groups	6	0.4099	0.0683			
Total:	11	7.2599				

**Table 8:** Statistical analysis of the results of determining the properties of fresh ICCM.

Name of the Property	*t*-Test (Empirical)	Critical t (at α = 0.05)	Degrees of Freedom (df)	*p*-Value	Effect Size (Cohen’s d)	Conclusion About the Difference Between Groups
ρ_1_	−2.666	2.571	5	0.045	−1.088 (big)	Significant
CSD	4.472			0.007	1.826 (big)	Significant
W	4.663			0.006	1.903 (big)	Significant

Statistically significant differences were observed between the ICCM groups without and with MS addition. MS content affects the soil stabilization properties of fresh ICCM.

**Table 9:** Changes in soil stabilization properties of fresh ICCM.

Properties Change	Content (%)				
	10	20	30	40	50
**C**					
∆$\rho_{1}$ (%)	−0.6	−1.8	−3.5	−5.3	−7.6
∆CSD (%)	−1.9	−9.3	−18.5	−25.9	−29.6
∆*W* (%)	−6.5	−14.5	−19.4	−27.4	−32.3
**C + MS**					
∆$\rho_{1}$ (%)	0	−1.2	−2.3	−2.9	−7.0
∆CSD (%)	−7.4	−13.0	−22.2	−29.6	−35.2
∆*W* (%)	−12.9	−19.4	−25.8	−35.5	−38.7

**Table 10:** Evaluation of variance for the results of determining the properties of hardened ICCM.

	Degrees of Freedom DF	Sum of Squares SS	Mean Square MS	F-Stat	*p*-Value	Conclusion
$\rho_{2}$						
Between Groups	5	0.0403	0.0081	60.4893	0.00005	Significant
Within Groups	6	0.0008	0.0001			
Total:	11	0.0411				
R_tb_						
Between Groups	5	8.7367	1.7473	69.9144	0.00003	Significant
Within Groups	6	0.15	0.025			
Total:	11	8.8866				
R						
Between Groups	5	428.2675	85.6535	41.1959	0.00014	Significant
Within Groups	6	12.4751	2.0792			
Total:	11	440.7426				

**Table 11:** Statistical analysis of the results of determining the properties of hardened ICCM.

Name of the Property	*t*-Test (Empirical)	Critical t (at α = 0.05)	Degrees of Freedom (df)	*p*-Value	Effect Size (Cohen’s d)	Conclusion About the Difference Between Groups
ρ_2_	−3.162	2.571	5	0.025	−1.291 (big)	Significant
R_tb_	−4.472			0.007	−1.826 (big)	Significant
R	−4.822			0.005	−1.969 (big)	Significant

**Table 12:** Transformations of the properties of hardened ICCM for soil stabilization depending on C and MS content.

Properties Change	Content (%)				
	10	20	30	40	50
**C**					
∆ρ_2_ (%)	−1.7	−3.4	−5.1	−7.4	−10.3
∆R_tb_ (%)	−10.4	−22.9	−37.5	−41.7	−54.2
∆R (%)	−13.2	−26.1	−41.3	−48.5	−60.1
**C + MS**					
∆$\rho_{2}$ (%)	−1.1	−2.9	−4.0	−5.7	−9.7
∆R_tb_ (%)	−6.3	−18.8	−31.3	−37.5	−47.9
∆R (%)	−5.6	−19.1	−33.3	−42.6	−51.8

**Table 13:** Carbon emissions per cubic meter.

Material	Carbon Equivalent (kgCO_2eq_/kg) [26,50]	Embodied Carbon (kgCO_2eq_)					
		Control Composition	10%	20%	30%	40%	50%
Portland cement	0.9235 (production+ logistics)	993.7	894.3	794.9	695.6	596.2	496.8
Clay	0.0042	0	0.5	0.9	1.4	1.8	2.3
Water	0	0	0	0	0	0	0
Microsilica	0.0098	0	0.21	0.21	0.21	0.21	0.21
Total CO_2_ emissions from material transport (excluding Portland cement) (kgCO_2eq_)		14					
Total CO_2_ emissions from the production of 1 m^3^ of mixture (kgCO_2eq_) [50]		12.3					
Sum:		1006.0	921.3	822.4	723.4	624.5	525.6

**Table 14:** Effect of Clay Raw Materials on the Properties of Cementitious Composites.

Reference	Modification Type	Result Achieved
[51]	Inclusion of 40% fired natural clay	Mixed cement mortars had improved durability characteristics
[52]	Replacement of part of the cement with 10–25% kaolinite clay	The strength properties of the composite are maintained and environmental performance is improved
[53]	Burnt limestone residual clay	Eco-friendly building solutions obtained
[54]	Clay cement + fly ash as a replacement for part of the Portland cement	Effective environmentally friendly solutions have been developed to stabilize chernozem soils
[55]	Red latosol clay 10–20%	Multi-purpose environmentally friendly solutions with optimal strength properties have been obtained
[56]	Replacement of part of the cement with up to 30% thermally activated clay	The solutions demonstrated improved durability and higher compressive strength
[57]	Replacement of part of the cement with up to 40% fired lime marl	Stable cement mortars with optimal strength properties were obtained
[58]	Modification of cement–clay mortars with MgO, xanthan gum, and carbomer	The increases in compressive and flexural strength of cement–clay mortars were 57.6% and 85.4%, respectively
[59]	Modification of cement mortars with silty clay powder	Including the addition of silty clay up to 10% instead of cement has little effect on the compressive strength of cement paste samples. At replacement levels greater than 30%, significant reductions in strength are observed.

## Data Availability

The original contributions presented in this study are included in the article. Further inquiries can be directed to the corresponding authors.

## Abbreviations

The following abbreviations are used in this manuscript:

C	Clay
ICCM	Injection cement–clay mortar
MS	Microsilica
PC	Portland cement
CSD	Cone spread diameter
